# Predictive performance and farmer satisfaction of *Jaɓnde*, a digital decision-support tool for dairy-cow ration formulation: results from field assessment in Burkina Faso

**DOI:** 10.1016/j.vas.2026.100837

**Published:** 2026-08-24

**Authors:** Songdah Désiré Ouattara, Ollo Sib, Mohamed Habibou Assouma, Souleymane Sanogo, Beh Traoré, Vinsoun Millogo, Valérie Marie Christiane Bougouma-Yaméogo, Eric Vall

**Affiliations:** a CIRAD, UMR SELMET, Bobo-Dioulasso, Burkina Faso; b SELMET, Univ Montpellier, CIRAD, INRAE, Institut Agro, Montpellier, France; c IDR, UNB, Bobo-Dioulasso, 01 BP 1091, Burkina Faso; d USPAE, CIRDES, Bobo-Dioulasso, 01 BP 454, Burkina Faso; e CIRAD, UMR SELMET, Dakar, Sénégal; f CIRAD, UMR SELMET, Nairobi, Kenya; g UNA, Abidjan, 02 BP801, Côte d'Ivoire; h CIRAD, UMR SELMET, F-34398, Montpellier, France

**Keywords:** Decision-support modelling, Milk production, Model assessment, Smallholder dairy systems, Tropical feeding systems

## Abstract

**Background and objectives:**

*Jaɓnde* is a locally adapted ration formulation tool for resource-constrained dairy systems in sub-Saharan Africa. The version used in this study was the Excel-based prototype installed on a laptop. A mobile version, now available for free on the Google Play Store and online (https://jabnde.cirad.fr/), is under testing. This preliminary, supervised field validation pursued three objectives: (i) to compare the technical and economic performance of zebu and crossbred cows under dairy farmer-implemented rations; (ii) to assess the implementation fidelity and the predictive performance of *Jaɓnde*; and (iii) to assess dairy farmer satisfaction with the use of *Jaɓnde.*

**Methodology:**

The tool was tested by 40 volunteer dairy farmers in the Bobo-Dioulasso milkshed (Burkina Faso) on 82 lactating cows (65 zebu, 17 crossbred). Implementation fidelity was assessed from the mean difference (predicted ration, R2 - actual ration, R3), and predictive performance from robust linear regression, reported overall and by cow type using R², the slope, and error indicators (mean absolute error, MAE; root mean square error, RMSE; mean bias error, MBE), complemented by Bland-Altman analyses. A structured survey assessed the satisfaction of dairy farmers.

**Results:**

Dairy farmers implemented R3 ration close to R2 (mean differences -0.37 to +0.04 kg GM/cow/day across feed types). Predicted and actual values were strongly associated for most variables (R² > 0.70), though weaker for milk production among crossbred cows; bias remained limited (MBE = -0.27 L/d/cow for milk production). Agreement on milk production was the most independent performance indicator. Crossbred cows produced more milk (10.6 vs 1.2 L/d/cow), whereas the relative change was greater in zebu cows (33%vs 8% increase). Over 90% of dairy farmers reported increased production and were satisfied.

**Contributions:**

This first field assessment is encouraging: under supervised conditions, farmers implemented *Jaɓnde*'s rations, predictive performance was promising for milk production, and satisfaction was high. Confirming its causal contribution and robustness will require controlled designs across more farms, seasons, and management systems.

## Highlights

• *Jaɓnde* supports least-cost ration formulation on smallholder dairy farms.
• *Jaɓnde* provides balanced rations tailored to tropical feed resources and dairy farmer constraints.
• *Jaɓnde’s* predictions were generally close to actual values.
• Participating dairy farmers found *Jaɓnde’s* advice trustworthy.

## Graphical abstract

**Figure:**
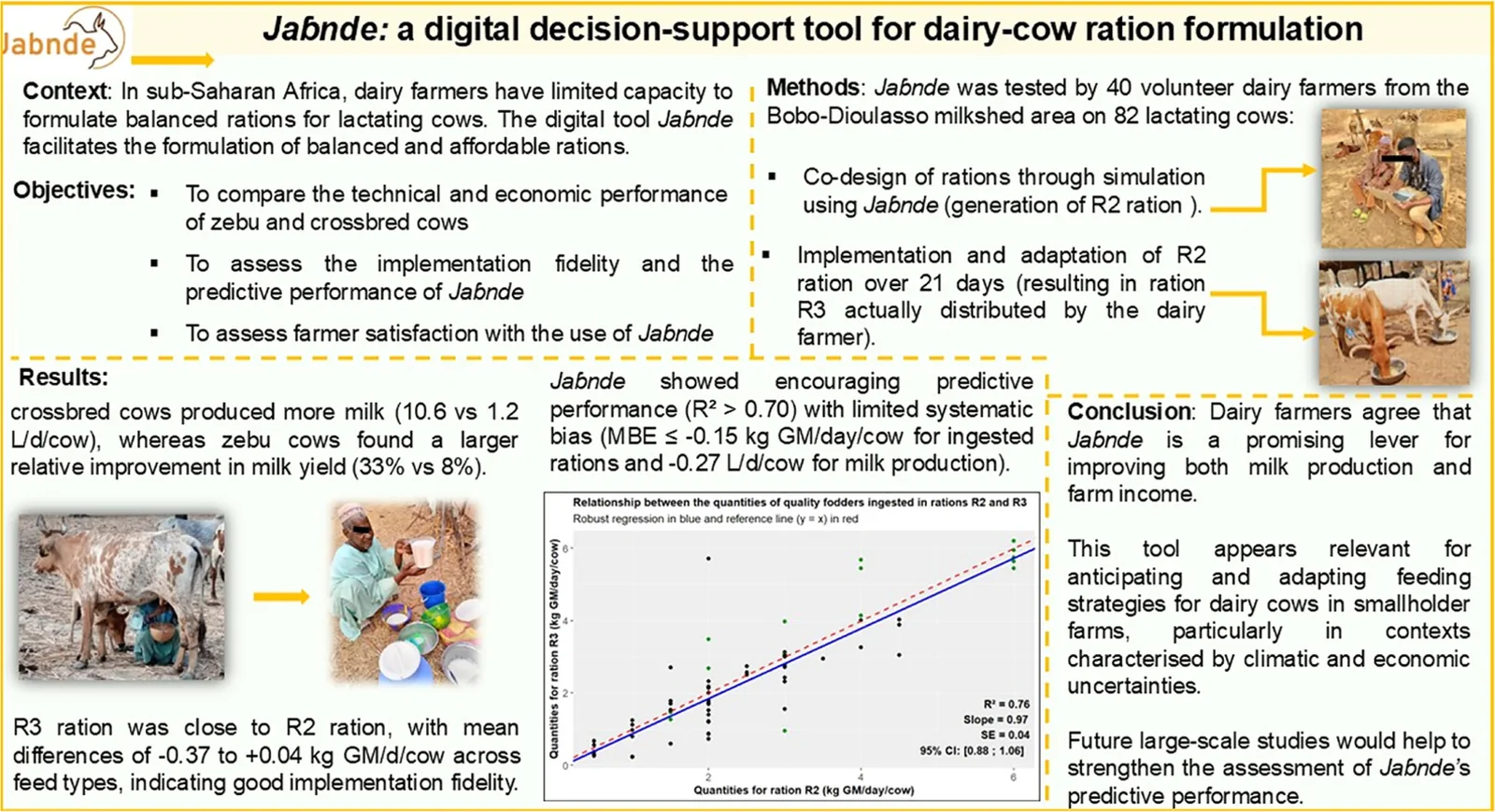

## 1 Introduction

Cow feed is the main item of dairy production expenditure and can easily be a limiting factor if not kept under control (Mijić et al., 2024; Molina-Flores et al., 2020; Sodré et al., 2022). In Sub-Saharan Africa, dairy farmers tend to follow an empirical approach when preparing rations for cows. This often leads to dietary deficiencies which affect milk yields, or, conversely, to wastage when too much feed is provided (Delma et al., 2016; Kassa et al., 2016a; Sib et al., 2018).

In Burkina Faso, as in most West African countries, milk production rests mainly on local suckler zebu cows and, to a lesser extent, crossbred cows. The latter are the result of crossbreeding between exotic dairy breeds and local breeds, or artificial insemination. They account for <10% of dairy herds (Kassa et al., 2016b; Ouédraogo et al., 2023; Sossouve et al., 2023; Vall et al., 2021). During the dry season, zebu cow milk production can range from 0.8 L/d/cow (Sib et al., 2017; Sodré et al., 2022) to 1.4 L/d/cow (Neya et al., 2025). Crossbreds produce between 7 L/d/cow (Vall et al., 2021) and 13 L/d/cow (Sib et al., 2017).

Tropical conditions constrain milk production: high temperatures and humidity, combined with marked seasonal rainfall, affect the animals' thermal comfort and the nutritional value of the available forage, thereby limiting their production potential (Moran, 2013; Yacoubou et al., 2024). Heat stress can be particularly detrimental to high-producing crossbred cows, which have higher metabolic demands and lower heat tolerance than local breeds; this may partly explain why they struggle to express their higher genetic potential for milk in such environments (Hanzen et al., 2024; Nsangou et al., 2021).

Cow feeding is generally based on three types of feed: natural rangeland vegetation, fodder (grown, crop co-products), and feed concentrates (Neya et al., 2023; Sib et al., 2017; Vall et al., 2021). However, these resources can vary widely in nutritional composition and cost, making it difficult to develop appropriate rations. This problem is compounded by seasonal fluctuations in the value of natural pastures and by the difficulty of accurately quantifying rangeland intake. In addition, dairy farmers have only limited technical reference points when it comes to estimating the actual nutritional requirements of their animals. As a result, they very often resort to trial and error approaches when formulating rations (Delma et al., 2016; Kassa et al., 2016a). This often produces unbalanced rations that are either insufficient, limiting yields, or excessive, resulting in feed wastage. These imbalances have economic consequences, such as lower profitability, and affect the health of the animals through increased mobilisation and depletion of their body reserves (Sib et al., 2017).

Considerable variability in feed resources and dairy farmers' relative inability to accurately assess their animals' actual feed requirements make it crucial to deploy a suitable advisory tool to help them formulate rations. Several studies have already attempted to address this issue by developing various rationing tools. These include **INRAtion V5 and RUMIN’AL** (INRA, 2007); **LIVSIM** (Rufino et al., 2009); **CLIFS** (Le Gal et al., 2013); **Altrop** (Delma et al., 2016); **Prodlait** (Sib et al., 2018), **NRC 2001** (NASEM, 2021), and **FAO Ration Tool** (FAO, 2025). Though these tools are generally suited to Sahelian conditions, they have a number of limitations. For example: (i) INRAtion V5 and RUMIN’AL do not include pasture grazing, (ii) LIVSIM is relatively complex to use, (iii) CLIFS is also relatively complex to use and too generic, (iv) Altrop is based on a limited number of feed resources and on imprecise pasture nutritional values depending on the season, (v) Prodlait considers only the coverage of energy requirements, (vi) NRC 2001 is not specifically adapted to dairy farming conditions in sub-Saharan Africa, (vii) FAO Ration Tool does not include pasture grazing and temperature. These limitations highlight the need for a ration formulation tool specifically adapted to the conditions of smallholder dairy farming in sub-Saharan Africa, which motivated the development of the ***Jaɓnde*** digital tool (Lecomte & Vall, 2022).

*Jaɓnde* formulates rations following two common feeding practices in Africa: open grazing and trough feeding. Breeds kept on African dairy farms (zebu, taurine, or crossbred) are taken into account. The tool incorporates a database of over 500 tropical feed resources (rangeland, fodder, and supplements). It also factors in the ambient temperature in order to fine-tune the calculation of feed and water requirements for cows. With *Jaɓnde*, all of these parameters can be incorporated in a more operational way to determine the most appropriate ration for dairy farmers to achieve their production targets. As such, *Jaɓnde* acts as a sustainability lever for dairy farms at the technical and economic levels through the formulation of balanced and affordable rations. At the social and environmental levels, it enhances dairy farmers’ decision-making independence while promoting greater use of local feed resources and better assessments of CH₄ and organic manure production levels.

An advisory and support tool designed for dairy farmers must indeed reconcile the nutritional balance of rations, economic profitability, and dairy unit output targets, while taking into account each cow's individual characteristics. Therefore, if they are to follow the recommendations drawn up by such a tool, dairy farmers must be able to trust it (Mijić et al., 2024; Yarashynskaya & Prus, 2022). In this context, two questions arise in relation to rationing tools: (i) how reliable are the rations simulated by these tools, i.e., how do they reflect reality under actual farming conditions, and (ii) can dairy farmers trust the results generated by such tools as a reliable basis for making feeding decisions?

The purpose of this study was to address these issues regarding the *Jaɓnde* tool, following a co-design field trial-based, iterative, and step-by-step methodological approach (Ouattara et al., 2025). More specifically, this study, conceived as a preliminary field validation of *Jaɓnde* under supervised conditions, pursued three objectives:

I. To characterise and compare the technical and economic performance of the two main cow types (zebu and crossbred) under dairy farmer-implemented rations (R3 ration);
II. To assess the tool itself along two complementary dimensions: the fidelity with which dairy farmers implemented the predicted ration (R2 ration), and the predictive performance of *Jaɓnde* for feed intake, milk production, ration cost, and gross profit margin; and
III. To assess dairy farmers’ satisfaction with the use of *Jaɓnde* for managing the feeding of their dairy cows.

## 2 Materials and methods

### 2.1 Study area and sampling strategy for volunteer dairy farmers

This study was carried out in the Bobo-Dioulasso milkshed area, which extends over a 50 km radius around the city of Bobo-Dioulasso. The local climate is Sudano-Sahelian, with annual rainfall of between 800 and 1100 mm and an alternating pattern of dry season (November to May) and rainy season (June to October). Rainfall is irregular, with uneven space-time distribution within the same crop year and from one crop year to the next. The average temperature ranges from 22 °C between November and February to 37 °C between March and May (Conseil régional des Hauts-Bassins, 2018). The Bobo-Dioulasso milkshed area constitutes a relevant case study because it reflects many of the constraints commonly faced by smallholder dairy systems in West Africa, including seasonal feed shortages, limited access to inputs, and reliance on local feed resources (Sib et al., 2020; Sodré, 2022; Vall et al., 2025a).

The study was carried out with 40 volunteer dairy farmers selected from the 300 dairy farmers of the Dairy Innovation Platform of Bobo-Dioulasso (Sib et al., 2023). These farmers form the backbone of the local dairy sector and play a central role in its operation and development, which makes the platform a relevant framework for studying dairy innovations under local production conditions. The herd sizes of these dairy farmers generally ranged from 3 to 180 head, with an average herd size ranging from 36 to 41 head per farm. The number of lactating cows varied from 2 to 47 head per farm (Orounladji et al., 2024; Sib et al., 2024). Lactating cows were predominantly zebu, whereas crossbred cows represented approximately 10% of the herd (Ouédraogo et al., 2023). During the dry season, farmers generally select 2 to 5 lactating cows for milk production. These cows received feed supplementation after grazing, typically consisting of quality fodder (legume haulms), coarse fodder (cereal residues), and feed concentrates. The availability of these resources is limited, and feed concentrates are often very expensive (Sib et al., 2017).

The 40 dairy farmers included in this study were selected according to the following criteria: (i) having sufficient stocks of quality fodder (legume haulms) and/or coarse fodder (cereal residues) to cover the trial phase; (ii) having lactating cows and a farm with easy access for monitoring purposes; and (iii) being willing to participate in the trial. These dairy farmers supplied seven milk collection centres (Bama, Bana, Benkadi, Belle Ville, Dafinso, Farakoba, and Yégueresso) affiliated to Bobo-Dioulasso's Dairy Innovation Platform. No statistical sample size calculation was used to determine the sample size. The sample size was limited to 40 dairy farmers due to financial and technical constraints associated with on-farm monitoring.

Because participation was voluntary and subject to specific logistical and technical criteria, the study population may not be statistically representative of all dairy farmers in the area: in particular, participating dairy farmers may have been more willing to experiment with new management practices than the wider dairy farming population. This voluntary participation was, however, a practical requirement for a supervised on-farm trial demanding close monitoring and reliable data recording throughout the trial period.

Dairy farmers were provided with technical support in formulating and testing feed rations for lactating cows during the dry seasons (February, March, and April) of 2024 and 2025. The *Jaɓnde* tool was thus used with a view to providing technical and economic advice on sound feeding practices for dairy farmers. The aim was to develop efficient (i.e., balanced and affordable) rations for the dry season. For dairy farmers, the dry season is indeed a critical time of year marked by a shortage of forage supplies (Sodré et al., 2022). On average, 2.05 ± 0.99 cows per dairy farmer were monitored during the trial, for a total of 82 rationed cows, including 17 crossbreds and 65 zebu cows. Each dairy farmer managed a single farm; the number of monitored cows varied from one farm to another, as follows: 1 cow on 8 farms, 2 cows on 26 farms, 3 cows on 5 farms, and 7 cows on 1 farm. Cows were selected by each farmer and fed according to their usual feeding practices. The cows selected were generally the most docile, in order to facilitate data collection. The number of cows selected for ration monitoring was limited by the fodder storage capacity available on each farm, as well as by our limited capacity to monitor multiple cows.

### 2.2 Presentation of the *Jaɓnde* tool

The prototype version of *Jaɓnde* used in this study (currently available in French only) was developed by CIRAD and INRAE within the standard Microsoft Office/Excel environment, with macros and calculation routines programmed in Visual Basic for field use on a basic laptop PC (Lecomte & Vall, 2022). This version of the tool is not freely available, and its use requires the support of a technical adviser. The mobile version, which is available free of charge on the Google Play Store and online (https://jabnde.cirad.fr/), is now available currently undergoing field testing.

**Fig. 1:**
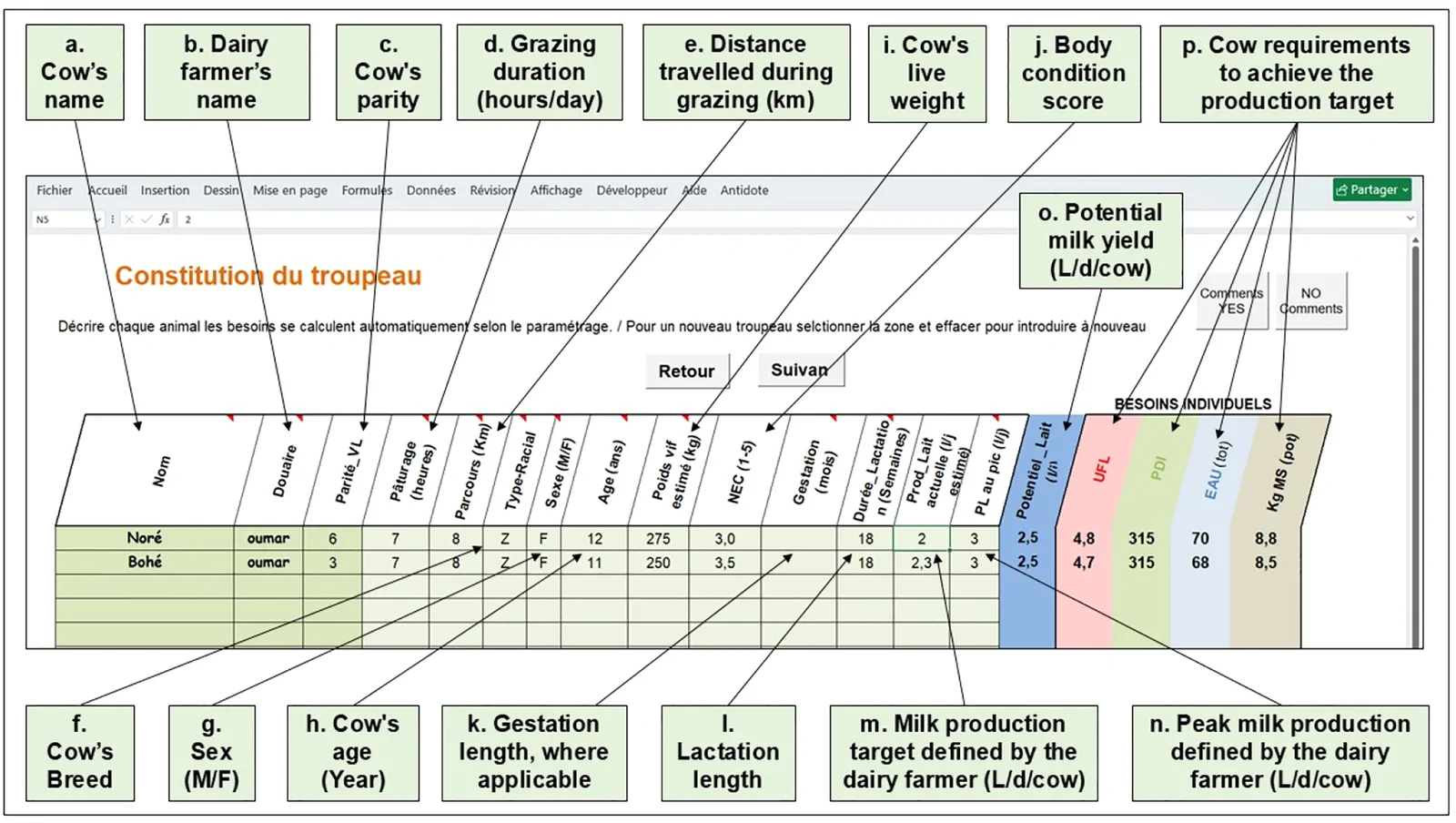
Input data required for calculating the feed requirements of cows to be rationed (*Jaɓnde*’s sheet 2).
• Sheet 3: it is dedicated to the selection of the feed resources available on the farm. It incorporates a database of over 500 tropical feed resources (rangeland, fodder, and supplements);
• Sheet 4: It allows the manual adjustment of the quantities of ration ingredients (forages and concentrates) and incorporates an automated optimisation function for individual supplementation under a least-cost constraint (this latter function was not used in the present study) (Fig. 2). A detailed description of the tool and its operation is provided in the technical manual, available at https://agritrop.cirad.fr/612645/ (Lecomte & Vall, 2022).

The tool was contextualised to reflect the general characteristics of dairy farming in Sub-Saharan Africa. *Jaɓnde* is organised into four main sheets:

• Sheet 1: it is dedicated to the concise characterisation of the farm (date, farm location, and ambient temperature);
• Sheet 2: it is used to record the characteristics of each cow to be rationed on the farm (Fig. 1);

**Fig. 2:**
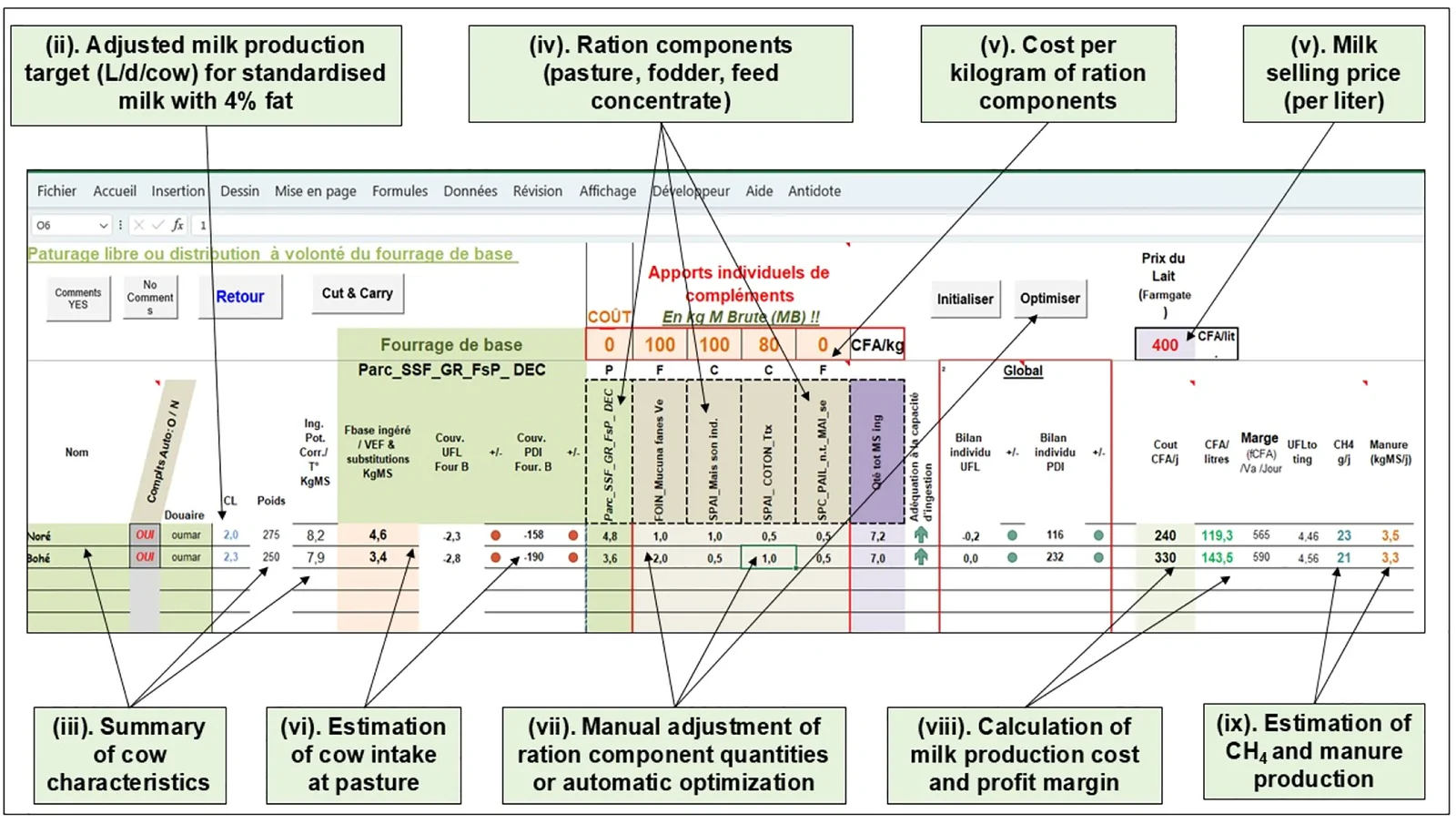
*Jaɓnde* operating diagram (*Jaɓnde*’s sheet 4).

So, *Jaɓnde* can be used to prepare individual rations for a herd of dairy cattle, using the following steps:

I. Given the ambient temperature
II. Given the dairy farmer’s production target;
III. Given the characteristics of lactating cows (genetic type, live weight, pregnancy status, lactation stage, peak milk production);
IV. Given the feeding practice followed (open grazing, trough feeding) and the ingredients that make up the ration (pasture, fodder, feed concentrates);
V. And given the price of these ingredients and the selling price of a litre of milk;
VI. The tool estimates the amount of natural pasture grass ingested per dairy cow put out to pasture;
VII. It can be used to manually adjust the quantities of the other ingredients in the ration (fodder and feed concentrates);
VIII. It calculates milk production costs and gross profit margins on feed costs;
IX. It estimates CH_4_ and organic manure production levels (Fig. 2).

Based on the cow’s maintenance and production requirements, *Jaɓnde* allows the simulation of different combinations of available feed quantities in order to meet energy requirements expressed in Milk Forage Unit (MFU) and protein requirements expressed in Intestinal Digestible Proteins (IDP). The tool provides an interface that displays the balance of MFU and IDP requirements coverage, as well as the ration cost, milk production cost per litre, daily gross margin, CH_4,_ and organic manure production. The ration cost and milk cost per litre are calculated exclusively based on feed prices and the quantities distributed. The gross margin is estimated by considering only the revenue from milk sales and the costs associated with the ration. For further details on the calculations and equations of the different *Jaɓnde* parameters, please refer to the tool’s technical manual (Lecomte & Vall, 2022).

All data presented on the figure are collected from the dairy farmer, except for cow requirements (p) and potential milk yield (o), which are automatically calculated by the tool based on the information entered. It should be noted, however, that points a, b, and j are not taken into account in the calculations. The ambient temperature entered in Sheet 1 is also taken into account by the tool when calculating the cows’ requirements. For further details, please refer to the tool’s technical manual (Lecomte & Vall, 2022).

For further details, please refer to the tool’s technical manual (Lecomte & Vall, 2022).

### 2.3 Ration co-design and implementation trial process

The field trial aimed at co-designing and implementing rations with dairy farmers was carried out in 4 stages.

#### 2.3.1 Stage 1: collecting reference and input data for *Jaɓnde*

The first stage of the trial consisted of collecting information on each dairy farm's feeding practices to characterise the cows selected by farmers for the trial. Using a questionnaire, data were collected each year between December and January on the following points: genetic type (zebu or crossbred), age, live weight (measured with a weighing tape), pregnancy status (number of months gestation where applicable), lactation week for each cow, presence of suckled calves, milk production target (predicted milk production per cow), milk production at peak lactation, fodder stock and available feed. An extra litre was added to milk quantities, reflecting the volume assumed to be consumed by the calf (Sossouve et al., 2023). The milk production target (predicted milk production per cow) was defined by each dairy farmer for each of their cows according to their own expectations and the cow’s previous milk production performance. This target was often adjusted based on the potential milk yield estimated by *Jaɓnde*. Although this farmer-defined target may carry a degree of subjectivity, and although the gap between the farmer’s expectation and realised yield is itself a source of variability, taking it into account was a deliberate methodological choice in this study. This approach is consistent with the participatory nature of *Jaɓnde*, designed as a decision-support tool enabling the formulation of rations adapted to the production objectives and constraints of dairy farmers. All reference and input data for *Jaɓnde* were collected by a single and same trained animal technician throughout the study.

#### 2.3.2 Stage 2: simulation-based ration co-design with *Jaɓnde*

This stage aimed to formulate a balanced ration based on dairy farmers' milk production targets (predicted milk production per cow monitored). For each cow, two rations were defined with the farmer:

• R1 (initial ration): it corresponds to the ration that the dairy farmer reported they would have provided during the last dry season. It represents an estimate of the quantities of feed that would have been offered per day per cow, as well as the grazing duration for each cow. Although this ration may involve a degree of subjectivity, it was primarily intended to provide an initial estimate of the quantities of feed ingredients to be entered into *Jaɓnde* and to serve as a starting point for co-designing a balanced and economically affordable ration with the dairy farmer. The objective was therefore not to assess the performance of this ration, but rather to use it as a starting point for the co-design process.
• R2 (predicted ration): it corresponds to the ration elaborated with the dairy farmer from the initial ration (R1), by adjusting the quantities of the different feeds and the grazing duration in order to meet the cow’s energy (MFU) and protein (IDP) requirements at a cost affordable to the farmer. R2 is the ration predicted by *Jaɓnde* and recommended to the dairy farmer. It was then implemented and assessed under real farming conditions during phase 3 of the study.

#### 2.3.3 Stage 3: R2 ration implementation and adaptation

During this stage, the aim was to characterise the rations actually implemented by dairy farmers, i.e., the R3 ration produced based on the R2 ration recommended to them. The trial lasted 21 days, including a 14-day adaptation period. Each dairy farmer was supported in implementing and adapting the R2 ration selected during the previous phase (Section 2.3.2).

During the adaptation period, farmers were assisted on the first two days of each of the first two weeks, i.e., Days 1, 2, 8, and 9 of the trial. They then implemented the ration on their own. Feed quantities to be distributed were measured with a spring scale (5 g accuracy) and calibrated using local measuring units (box, rope, plastic bag, hollow container, etc.). This gave dairy farmers an idea of how much feed to distribute. During this period, adjustments to R2 rations were made whenever necessary, in close cooperation with dairy farmers, to ensure they were suited to actual livestock farming conditions.

The final ration recommended to dairy farmers is referred to as R2 (predicted ration), and the ration actually implemented by dairy farmers is called R3 (actual ration). Variables related to the recommended R2 ration are identified by the prefix “predicted”, whereas variables related to the R3 ration are identified by the prefix “actual”. Table 1 presents a descriptive summary of all the rations.

**Table 1:** Descriptive summary of the different rations.

Different rations	Descriptions
R1 (initial ration)	It corresponds to the ration that the dairy farmer reported they would have provided during the last dry season. It represents an estimate of the quantities of feed that would have been offered per day per cow, as well as the grazing duration for each cow. Although this ration may involve a degree of subjectivity, it was primarily intended to provide an initial estimate of the quantities of feed ingredients to be entered into Jaɓnde and to serve as a starting point for co-designing a balanced and economically affordable ration with the dairy farmer. The objective was therefore not to assess the performance of this ration, but rather to use it as a starting point for the co-design process.
R2 (predicted ration)	It corresponds to the ration elaborated with the dairy farmer from the initial ration (R1), by adjusting the quantities of the different feeds and the grazing duration in order to meet the cow’s energy (MFU) and protein (IDP) requirements at a cost affordable to the farmer. R2 is the ration predicted by *Jaɓnde* and recommended to the dairy farmer. It was then implemented and assessed under real farming conditions during phase 3 of the study.
R3 (actual ration)	It corresponds to the ration actually implemented by the dairy farmer based on ration R2.

Data collection lasted 10 days from Day 12. However, only data from the last 7 days was used to analyse the results. The following data were collected using a daily follow-up sheet: (i) feed quantities actually delivered to cows; (ii) feed intake levels; (iii) daily grazing time; (iv) milked quantities per cow per day, measured using a measuring cup.

#### 2.3.4 Stage 4: dairy farmer satisfaction survey on *Jaɓnde* forecasts

Once the trial phase was over, a satisfaction survey was carried out among dairy farmers. This survey looked at their level of satisfaction with the impact of implementing R2 ration on their cows' milk production and their income. Their perception of rationing via *Jaɓnde* as a lever for improving cow milk production and income was also assessed.

Variables collected to assess dairy farmers' satisfaction levels for each cow (82 cows) were as follows: (i) satisfaction with actual milk production levels; (ii) level of change in milk production relative to milk production on day 1 of the trial; (iii) satisfaction with achieved gross profit margin; (iv) reasons for achieving milk production targets (multiple-choice question); (v) reasons for not achieving milk production targets (multiple-choice question).

Variables collected to assess dairy farmers' satisfaction levels per dairy farmer (40 dairy farmers) were as follows: (i) difference between milk production from cows rationed with *Jaɓnde* versus those not rationed with *Jaɓnde* at farm level, as perceived by the dairy farmers, since no formal control group was included in the study; (ii) impact of the trial on cow feeding practices (multiple-choice question); and (iii) use of *Jaɓnde* as a lever for improving cow milk production and income.

All data collected during the trial (Section 2.3) were digitised on the KoboToolbox platform and deployed on the KoboCollect tool for data collection (Nampa et al., 2020). Using the KoboCollect application, they were deployed on a tablet. The data collected via the tablet were then sent to the KoboToolbox platform before being exported as an Excel file for analysis.

### 2.4 Data processing and analysis

All collected data, as well as data calculated using *Jaɓnde*, were entered into an Excel spreadsheet and exported to R software version 4.3.3 (R Core Team, 2024) for statistical analysis. Key quantitative variables collected and developed by *Jaɓnde* are shown in Table 2. In order to obtain an independent unit of analysis, daily variables were averaged per cow over the last 7 days (Section 2.3.3). Analyses were conducted at the cow level and did not explicitly account for the hierarchical structure of the data (cows nested within farms). For each cow, the variables included in the analyses corresponded to the average values recorded over the final seven days of monitoring. Daily measurements were not entered individually; therefore, each cow contributed a single observation to the analyses, which reduced issues related to repeated daily measurements.

**Table 2:** Key quantitative variables collected or calculated by *Jaɓnde*.

Variables	Units	Descriptions	Collection methods
Ambient temperature	°C	Mean temperature in the study area during the trial period *(Jaɓnde* input data)	Source: https://fr.weatherspark.com/y/35126/M %C3%A9t%C3%A9o-moyenne-%C3%A0-Bobo-Dioulasso-Burkina-Faso-tout-au-long-de-l'ann%C3%A9e
Cow's age	Year	*Jaɓnde* input data	Survey
Cow's live weight	kg	*Jaɓnde* input data	Measured with a weighing tape
Cow's parity	Number of calvings	*Jaɓnde* input data	Survey
Body Condition Score (BCS)	Score/cow	This data is purely descriptive and is not used in the calculation of the cow’s requirements.	Assignment of a score from 1 to 5 per observation based on a grid devised by Vall and Bayala (2004) and Vall et al. (2025b)
Lactation length	Week/cow	*Jaɓnde* input data	Survey
Milk production target (Predicted milk production)	L/d/cow	The milk production target was defined by each dairy farmer for each of their cows according to their own expectations and was often adjusted based on the potential milk yield estimated by *Jaɓnde*. Corresponding to the quantity of milk to be milked. *(Jaɓnde* input data)	Survey
Milk Forage Unit (MFU) requirements	MFU/d/cow	Total quantity of energy, expressed as MFU, required to cover daily needs for upkeep, mobility, gestation, body reserve replenishment, and observed lactation. For further details on the calculations and equations, please refer to the tool’s technical manual (Lecomte & Vall, 2022).	Calculated by *Jaɓnde* from input data
Intestinal Digestible Protein (IDP) requirements	g IDP/d/cow	Total quantity of proteins, expressed as IDP required to cover daily needs for upkeep, gestation, body reserve replenishment, and observed lactation. For further details on the calculations and equations, please refer to the tool’s technical manual (Lecomte & Vall, 2022).	Calculated by *Jaɓnde* from input data
Total Dry Matter (DM) requirements	kg DM/d/cow	Quantity of dry matter that cows would ideally be able to ingest given their weight, milk production level, and ambient temperature. For further details on the calculations and equations, please refer to the tool’s technical manual (Lecomte & Vall, 2022).	Calculated by *Jaɓnde* from input data
Total water requirements	L/d/cow	Total quantity of water required daily by cows, allowing for variations linked to zebu or taurine type and the impact of average temperatures above 25 °C. Some of this water is found in gross feed matter, in particular green fodder. For further details on the calculations and equations, please refer to the tool’s technical manual (Lecomte & Vall, 2022).	Calculated by *Jaɓnde* from input data
Quantity of quality fodder	kg GM/d/cow	∑ Quantity of cowpea haulms+ quantity of peanut haulms+ quantity of *mucuna*+ quantity of potato haulms (used by a single dairy farmer)	Measured during the trial
Quality fodder refusal rate	%	[(Quantity of quality fodder distributed - quantity of quality fodder ingested) / quantity of quality fodder distributed] × 100	Calculated
Quantity of coarse fodder	kg GM/d/cow	∑ Quantity of maize straw+ quantity of sorghum straw+ quantity of rice straw+ other types of coarse fodder. Other types of coarse fodder were used, but not extensively: bush hay by only four dairy farmers, panicum by one dairy farmer, and brachiaria by one dairy farmer	Measured during the trial
Coarse fodder refusal rate	%	[(Quantity of coarse fodder distributed - quantity of coarse fodder ingested) / quantity of coarse fodder distributed] × 100	Calculated
Quantity of feed concentrates	kg GM/d/cow	∑ Quantity of maize bran+ quantity of rice bran+ quantity of cottonseed cake+ quantity of brewer's spent grain (only for crossbreds)	Measured during the trial
Feed concentrates refusal rate	%	[(Quantity of feed concentrates distributed - quantity of feed concentrates ingested) / quantity of feed concentrates distributed] × 100	Calculated
IDP intake from ingested rations	g IDP/d/cow	∑ Ingested quantity of each feed in the ration **×** feed DM coefficient **×** feed IDP value For further details on the feed DM coefficient and its IDP value, please refer to the tool’s technical manual (Lecomte & Vall, 2022).	Calculated
MFU intake from ingested rations	MFU/d/cow	∑ Ingested quantity of each feed in the ration **×** feed DM coefficient **×** feed MFU value For further details on the feed DM coefficient and its MFU value, please refer to the tool’s technical manual (Lecomte & Vall, 2022).	Calculated
Milk production on Day 1	L/d/cow	Cow's milk production before R3 ration distribution, corresponding to the quantity milked by the herdsman.	Measured on Day 1 of the trial
Actual milk production	L/d/cow	Average production over the last 7 days of the trial, corresponding to the quantity milked by the herdsman.	Measured during the trial
Maximum actual milk production	L/d/cow	Maximum production over the last 7 days of the trial, corresponding to the quantity milked by the herdsman.	Measured during the trial
Actual cost per litre of milk	FCFA/L/d/cow	(Average cost of R3 ration distributed over the last 7 days of the trial) **/** (Actual milk production)	Calculated
Predicted gross profit margin	FCFA/d/cow	(Predicted milk quantity **×** selling price per predicted litre of milk) **-** (cost of R2 ration distributed)	Since R2 rations developed by *Jaɓnde* are assumed to be fully ingested, we calculated the R3 ration refusal rate and applied it to the R2 ration to obtain the R2 ration distributed. This enabled us to calculate the cost of the R2 ration distributed.
Actual gross profit margin	FCFA/d/cow	(Actual milk production **×** selling price per litre of milk) **-** (average cost of R3 ration distributed during the last 7 days of the trial)	Calculated
Potential CH_4_ production	g/d/cow	Estimate of daily methane emissions as a direct function of the quantity of organic matter fermented in a cow's rumen. For further details on the calculations and equations, please refer to the tool’s technical manual (Lecomte & Vall, 2022).	Calculated by *Jaɓnde* from input data. The tool does not calculate emissions from cows kept in stalls.
Potential organic manure production	kg DM/d/cow	Estimate of the quantity of undigested dry matter released by cows. For further details on the calculations and equations, please refer to the tool’s technical manual (Lecomte & Vall, 2022).	Calculated by *Jaɓnde* from input data

All reference and input data for *Jaɓnde* were collected by a single and same trained animal technician throughout the study; 1 Euro= 655.957 FCFA; MFU= Milk Forage Unit; IDP= Intestinal Digestible Proteins; kg DM= Kilogram of Dry Matter; kg GM= Kilogram of Gross Matter; BCS= Body Condition Score; d= day; L= Litre; g= gram.

To characterise and compare the technical and economic performance of the two main cow types (zebu and crossbred) under dairy farmer-implemented rations (R3 ration), data normality was checked using the Shapiro–Wilk test. Variance homogeneity was assessed using Levene's test. When normality was met, but variances were unequal, a Welch's *t*-test was performed. When the assumption of normality was not met, the non-parametric Wilcoxon test was used. The significance level was set at p < 0.05 for all tests. Variables considered to compare the performance between zebu and crossbred cows were as follows: quality fodder (54 zebus cows vs 16 crossbred cows); coarse fodder (39 zebus cows vs 7 crossbred cows); feed concentrates (63 zebus cows vs 17 crossbred cows); grazing time (55 zebus cows vs 12 crossbred cows); IDP intake from ingested ration (65 zebus cows vs 17 crossbred cows); MFU intake from ingested ration (65 zebus cows vs 17 crossbred cows); milk productions (65 zebus cows vs 17 crossbred cows); cost per litre of milk (65 zebus cows vs 17 crossbred cows); gross profit margin (65 zebus cows vs 17 crossbred cows); Potential CH_4_ production (55 zebus cows vs 12 crossbred cows); Potential organic manure production (65 zebus cows vs 17 crossbred cows). The variables did not apply to all cows.

To assess *Jaɓnde* along two complementary dimensions: the fidelity with which dairy farmers implemented the predicted rations (R2) and the predictive performance of tool, the key variables considered for these analysis were as follows: (i) quantity of quality fodder ingested (kg GM/d/cow); (ii) quantity of coarse fodder ingested (kg GM/d/cow); (iii) quantity of feed concentrates ingested (kg GM/d/cow); (iv) IDP intake from ingested ration (g IDP/d/cow); (v) MFU intake from ingested ration (MFU/d/cow); (vi) milk production (L/d/cow); (vii) total cost of distributed ration (F CFA/d/cow), and (viii) gross profit margin on the cost of distributed ration (F CFA/d/cow). No missing values were recorded for any of the variables considered. All 82 cows were included in the analyses, save for a few variables: quality fodder (70 cows), coarse fodder (43 cows), and feed concentrates (78 cows), which did not apply to all cows.

To assess the fidelity with which dairy farmers implemented the R2 ration, the mean difference between the actual ration (R3) and the R2 ration (R3 - R2) was calculated for each variable listed above, both for the entire sample of 82 cows and according to cow type (zebu and crossbred cows).

To assess the predictive performance of *Jaɓnde*, the associations between R2 and R3 rations were analysed for all cows and by cow type. For each variable listed above, correlations between the values predicted by *Jaɓnde* (R2 ration) and those recorded in the R3 ration produced by dairy farmers were analysed. A robust linear regression was performed to reduce the influence of outliers likely to distort the ordinary least squares estimates using the following equation:

**Y** = **β_0_** + **β_1_X** + **ε**. Where **Y**= dependent variable (the outcome); **β_0_**= intercept_;_ **β_1_**= model slope; **X**= explanatory variable (the expected outcome), and **ε**= random error.

The robust model was adjusted using the robust linear model (*rlm*) function of the *MASS package* under R software, based on Huber's M estimator, which assigns lower weights to readings with large residuals. This approach provides a more reliable estimate of the slope, together with its standard error and t-value (statistical significance of the slope).

Two hypotheses regarding the regression slope (β₁) were examined. The first (H₀: β₁= 0) tested the existence of an association between predicted and actual values, with the test statistic computed as t-value= β̂_1_ / SE (β̂_1_). The second (H₀: β₁= 1) assessed the departure from perfect agreement between predicted and actual values, with t-value= (β̂_1_ − 1) / SE (β̂_1_). For each hypothesis, a two-sided *p*-value was derived from Student's t-distribution with (n − 2) degrees of freedom, where n is the number of observations. A 95% confidence interval (CI) for the slope was also estimated as CI= β̂_1_ ± 1.96 × SE (β̂_1_). Because the *rlm* function does not provide an exact theoretical basis for *p*-values, the *p*-values and confidence intervals are treated as approximate, being derived from the standard errors of the robust regression and from Student's t-distribution.

Given that robust models do not directly provide a coefficient of determination, a pseudo-R² was calculated as the square of the correlation between actual values and the model's predicted values. This pseudo-R² is a measure of goodness of fit that helps to assess the strength of the modelled relationship in a robust framework (Finger & Hediger, 2007; Yu & Yao, 2017). Three complementary indicators were calculated to assess the predictive performance of the robust regression model: the mean absolute error (MAE), the root mean square error (RMSE), and the mean bias error (MBE). These indicators were calculated from the residuals of the robust regression model, using the actual values and the corresponding values predicted by the model. The MAE was defined as the mean of the absolute differences between actual and predicted values, whereas the RMSE corresponded to the square root of the mean squared differences between these values. The MBE represented the mean difference between actual and predicted values.

Given the limited number of crossbred cows included in the study (n = 17), the predictive performance estimates for this subgroup, particularly for milk production, should be interpreted with caution.

In addition, Bland-Altman plots were generated to assess the agreement between actual and predicted values, including the 95% limits of agreement for milk production and gross profit margin.

Robust linear regression was preferred to ordinary least squares regression because the data did not fully satisfy the assumptions of the classical linear model. The presence of influential observations and violations of normality and homoscedasticity assumptions justified the use of a robust approach, which provides more reliable estimates under these conditions.

The assessment of dairy farmers' satisfaction was based on the calculation of relative frequencies of responses to the survey, expressed as a percentage (%) for the modalities of each variable (section 2.3.4). The analysis provided an opportunity to assess whether dairy farmers were satisfied or not per rationed cow. For variables for which data were collected at cow level, percentages were calculated by cow type. For variables recorded at the dairy farmer level, percentages were calculated based on the total number of surveyed dairy farmers. For variables derived from multiple-choice questions administered at the cow level, percentages corresponding to each modality, calculated by cow type, were standardised on a 0 - 100% scale. In contrast, for the variable “impact of the trial on cow feeding practices”, recorded at the dairy farmer level, percentages were calculated for each modality based on the number of dairy farmers who selected it.

## 3 Results

### 3.1 Characteristics and technical-economic performance of zebu and crossbred cows

#### 3.1.1 Cow characteristics and requirements

Overall, crossbred cows differed from zebu cows by having significantly higher characteristics and requirements (P < 0.05). In contrast, parity number and lactation length did not differ significantly between the two cow types (Table 3).

**Table 3:** Rationed cows' characteristics and requirements.

Cows' characteristics and requirements	Crossbred cows	Zebu cows	P-value
Number of cows	17	65	
Age (years/cow)	5 ± 1	7 ± 2	< 0.05
Weight (kg/cow)	434 ± 86	248 ± 45	< 0.05
Parity number per cow	3 ± 1	3 ± 1	NS
Body condition score (BCS) per cow	3.8 ± 0.4	3.3 ± 0.4	< 0.05
Lactation length (weeks/cow)	15 ± 12	17 ± 9	NS
Predicted milk production (L/d/cow)	10.9 ± 3.7	1.7 ± 0.7	< 0.05
MFU requirements (MFU/d/cow)	11.8 ± 2.3	5.2 ± 0.8	< 0.05
IDP requirements (g IDP/d/cow)	912 ± 176	336 ± 46	< 0.05
Total DM requirements (kg DM/d/cow)	13.58 ± 1.6	8.5 ± 0.8	< 0.05
Total water requirements (L/d/cow)	110 ± 22	65 ± 8	< 0.05

MFU= Milk Forage Unit; IDP= Intestinal Digestible Proteins; kg DM= Kilogram of Dry Matter; kg GM= Kilogram of Gross Matter; BCS= Body Condition Score; L= Litre; kg= kilogram; d= day; P < 0.05 indicates a significant difference; NS= not significantly different (P > 0.05).

The average age of crossbreds was 5 years, with a live weight of 434 ± 86 kg/cow and an average body condition score close to 4. The average parity number was 3, and the average lactation length was 15 weeks. For a predicted milk production of 10.9 ± 3.7 L/d/cow, the cows' upkeep and production requirements were 11.8 ± 2.3 MFU/d/cow and 912 ± 176 g IDP/d/cow.

Zebu cows had an average age of 7 years, with a live weight of 248 ± 45 kg/cow and an average body condition score of 3. The average parity number was 3, and the average lactation length was 17 weeks. For a predicted milk production of 1.7 ± 0.7 L/d/cow, the cows' upkeep and production requirements were 5.2 ± 0.8 MFU/d/cow and 336 ± 46 g IDP/d/cow (Table 3:).

#### 3.1.2 Feed intake, milk production, cost, and margin by cow type

The following results describe the performance actually achieved under dairy farmer-implemented R3 ration; the accuracy with which *Jaɓnde* predicted these values is addressed separately under the assessment of the tool (section 3.2.1). The estimates of enteric methane (CH₄) production presented in this study (Table 4) should be interpreted with caution, as they have not been validated against direct measurements of CH₄ emissions (Table 2).

**Table 4:** R3 rations' characteristics and technical and economic performance of crossbreds and zebu cows.

Variables	Crossbred cows	Zebu cows	P-value
Quality fodder distributed (kg GM/d/cow)	4.3 ± 1.6	2.2 ± 1.2	< 0.05
Quality fodder ingested (kg GM/d/cow)	4.0 ± 1.8	1.9 ± 1.2	< 0.05
Quality fodder refusal rate (%)	8 ± 18	18 ± 22	NS
Coarse fodder distributed (kg GM/d/cow)	5.6 ± 2.9	2.4 ± 2.3	< 0.05
Coarse fodder ingested (kg GM/d/cow)	4.1 ± 2.6	1.6 ± 1.3	< 0.05
Coarse fodder refusal rate (%)	34 ± 27	25 ± 25	NS
Feed concentrates distributed (kg GM/d/cow)	11.2 ± 2.9	2.7 ± 0.8	< 0.05
Feed concentrates ingested (kg GM/d/cow)	11.1 ± 3.0	2.6 ± 0.9	< 0.05
Feed concentrates refusal rate (%)	1 ± 4	4 ± 0.1	NS
Grazing time (h/d)	3 ± 1	9 ± 3	< 0.05
IDP intake from ingested ration (g IDP/d/cow)	1138 ± 267	473 ± 120	< 0.05
MFU intake from ingested ration (MFU/d/cow)	12 ± 2	5 ± 1	< 0.05
Milk production on Day 1 (L/d/cow)	9.8 ± 3.2	0.9 ± 0.4	< 0.05
Actual milk production (L/d/cow)	10.6 ± 1.7	1.2 ± 0.5	< 0.05
Maximum actual milk production (L/d/cow)	12.7 ± 1.8	1.4 ± 0.5	< 0.05
Actual cost per litre of milk (F CFA/L/d/cow)	138 ± 42	333 ± 161	< 0.05
Actual gross profit margin (F CFA/d/cow)	3142 ± 720	158 ± 224	< 0.05
Potential CH_4_ production (g/d/cow)	36 ± 7	20 ± 3	< 0.05
Potential organic manure production (kg DM/d/cow)	6 ± 1	4 ± 1	< 0.05

kg GM= Kilogram of Gross Matter; d= day; kg DM= Kilogram of Dry Matter; MFU= Milk Forage Unit; IDP= Intestinal Digestible Proteins; 1 Euro= 655.957 F CFA; L= Litre; g= gram; P < 0.05 indicates a significant difference; NS= not significantly different (P > 0.05). Actual cost per litre of milk= estimation of the cost per litre of milk produced, considering only the feed cost of the R3 ration distributed and the recorded actual milk production; Actual gross profit margins= gross margin estimated considering only milk sales revenue and R3 ration feed costs.

Feed quantities for R3 rations distributed and ingested by crossbred cows were higher (P < 0.05) than those of zebu cows (Table 4). Among crossbreds, the ingested R3 ration consisted of 4.0 ± 1.8, 4.1 ± 2.6, and 11.1 ± 3.0 kg GM/d/cow, respectively, for quality fodder, coarse fodder, and feed concentrates. For zebu cows, the ingested R3 ration consisted of 1.9 ± 1.2, 1.6 ± 1.3, and 2.6 ± 0.9 kg GM/d/cow, respectively, for quality fodder, coarse fodder, and feed concentrates. Zebu cows spent more time grazing (9 ± 3 h/d) than crossbreds (3 ± 1 h/d).

IDP and MFU intakes from R3 rations ingested by crossbred cows were also higher (P < 0.05) than those of zebu cows. Among crossbred cows, the IDP intake from the ingested ration was 1138 ± 267 g IDP/d/cow, and the MFU intake was 12 ± 2 MFU/d/cow. For zebu cows, the IDP intake from the ration was 473 ± 120 g IDP/d/cow, and the MFU intake was 5 ± 1 MFU/d/cow (Table 4).

Crossbred cows' milk production was higher (P < 0.05) than that of zebu cows. The same applies to production costs per litre of milk and actual gross profit margins. Among crossbred cows, actual milk production increased by 8%, from 9.8 ± 3.2 L/d/cow on Day 1 of the trial to 10.6 ± 1.7 L/d/cow. Maximum actual milk production was 12.7 ± 1.8 L/d/cow, i.e., a rise of 30%. For zebu cows, actual milk production increased by 33%, from 0.9 ± 0.4 L/d/cow on Day 1 of the trial to 1.2 ± 0.5 L/d/cow. Maximum actual milk production was 1.4 ± 0.5 L/d/cow, i.e., an increase of 56% (Table 4).

The actual cost per litre of milk was 138 ± 42 and 333 ± 161 F CFA/L/d/cow for crossbreds and zebu cows, respectively. As for the actual gross profit margin, it was 3142 ± 720 and 158 ± 224 F CFA/L/d/cow, respectively, for crossbreds and zebu cows. Crossbred cows' potential CH_4_ production (36 g/d/cow) was higher (P < 0.05) than that of zebu cows (20 g/d/cow). The same applies to potential organic manure production (Table 4).

### 3.2 Implementation fidelity of R3 ration and predictive performance of *Jaɓnde*

Whereas the previous section compared the actual performance achieved by the two cow types, the present section assesses how accurately *Jaɓnde* predicted these outcomes.

#### 3.2.1 Farmers' ability to implement the predicted rations

The R3 ration implemented by the dairy farmers was close to the predicted ration (R2). The mean differences between R3 and R2 ration ingested (R3 - R2) were −0.10 kg GM/d/cow for quality fodder, −0.37 kg GM/d/cow for coarse fodder, and +0.04 kg GM/d/cow for feed concentrates. Among zebu cows, these differences were −0.19, −0.45, and +0.01 kg GM/d/cow, respectively, whereas for crossbred cows they were +0.19, +0.15, and +0.14 kg GM/d/cow.

The IDP and MFU intake from the R3 ration ingested were also close to those of the R2 ration ingested. Among zebu cows, the mean differences were −14.6 *g* IDP/d/cow and −0.14 MFU/d/cow, respectively, whereas for crossbred cows, the mean differences were +23.4 *g* IDP/d/cow and +0.22 MFU/d/cow.

In terms of total costs of rations, the mean difference between R2 and R3 ration distributed was −39.4 F CFA /d/cow for zebu cows and +39 F CFA /d/cow for crossbred cows.

With regard to productive performance, the mean difference between predicted and actual milk production was −0.52 L/d/cow for zebu cows and −0.29 L/d/cow for crossbred cows. The mean difference between the predicted and actual gross profit margin was −185 F CFA/d/cow for zebu cows and −207 F CFA/d/cow for crossbred cows (Table 5).

**Table 5:** Characteristics of R2 and R3 rations, production performance, and mean differences.

Variables		Number of cows	Predicted ration (R2)	Actual ration (R3)	Mean difference (R3 - R2)
Quality fodder ingested (kg GM/d/cow)	All the cows	70	2.47 ± 1.44	2.37 ± 1.60	−0.10
	Zebu cows	54	2.07 ± 1.08	1.88 ± 1.17	−0.19
	Crossbred cows	16	3.81 ± 1.71	4.01 ± 1.79	+0.19
Coarse fodder ingested (kg GM/d/cow)	All the cows	43	2.31 ± 2.12	1.95 ± 1.80	−0.37
	Zebu cows	37	1.95 ± 1.73	1.49 ± 1.26	−0.45
	Crossbred cows	6	4.58 ± 3.01	4.74 ± 2.19	+0.15
Feed concentrates ingested (kg GM/d/cow)	All the cows	78	4.46 ± 3.84	4.49 ± 3.86	+0.04
	Zebu cows	61	2.64 ± 0.80	2.65 ± 0.89	+0.01
	Crossbred cows	17	11.00 ± 3.28	11.10 ± 3.03	+0.14
IDP intake from ingested ration (g IDP/d/cow)	All the cows	82	617.12 ± 291.67	610.42 ± 314.78	−6.69
	Zebu cows	65	487 ± 108	473 ± 120	−14.6
	Crossbred cows	17	1114 ± 230	1138 ± 267	+23.4
MFU intake from ingested ration (MFU/d/cow)	All the cows	82	6.43 ± 2.75	6.37 ± 2.91	−0.06
	Zebu cows	65	5.15 ± 0.80	5.01 ± 0.88	−0.14
	Crossbred cows	17	11.30 ± 1.91	11.50 ± 1.97	+0.22
Milk production (L/J/cow)	All the cows	82	3.63 ± 4.12	3.16 ± 3.92	−0.47
	Zebu cows	65	1.73 ± 0.66	1.21 ± 0.48	−0.52
	Crossbred cows	17	10.90 ± 3.67	10.60 ± 1.70	−0.29
Total cost of rations distributed (F CFA /d/cow)	All the cows	82	612 ± 485	589 ± 514	−23.24
	Zebu cows	65	402 ± 185	363 ± 162	−39.4
	Crossbred cows	17	1416 ± 432	1454 ± 477	+39
Gross profit margins (F CFA /d/cow)	All the cows	82	967 ± 1414	777 ± 1274	−190
	Zebu cows	65	344 ± 261	158 ± 224	−185
	Crossbred cows	17	3349 ± 1495	3142 ± 720	−207

kg GM= Kilogram of Gross Matter; d= day; MFU= Milk Forage Unit; IDP= Intestinal Digestible Proteins; 1 Euro= 655.957 F CFA; L= Litre; g= gram; R2= final ration recommended to dairy farmers (predicted ration); R3= ration actually implemented by dairy farmers (actual ration); Total cost of rations distributed= estimated ration cost based exclusively on feed prices and distributed quantities; Gross profit margins= gross margin estimated considering only milk sales revenue and feed costs; Milk production= actual milk production for R3 ration and predicted milk production for R2 ration.

#### 3.2.2 Predictive performance of *Jaɓnde*

The agreement between the R2 and R3 rations, as well as the predictive performance of *Jaɓnde,* are presented below for feed intake, milk production, distributed ration cost and gross profit margin. The results are presented both for all cows combined and separately by cow type, and are summarised in Table 6.

**Table 6:** Key indicators for assessing *Jaɓnde*'s predictive performance based on the robust regression model.

Variables		Number of cows	pseudo-R² (R²)	Regression Slope (β1)	Standard error of the slope	Slope CI (95%)	t-value (β1 = 1)	p-value (β1 = 1)	t-value (β1 = 0)	p-value (β1 = 0)	Mean absolute error (MAE)	Root mean square error (RMSE)	Mean bias error (MBE)
Quality fodder ingested (kg GM/d/cow)	All the cows	70	0.76	0.97	0.04	[0.88; 1.06]	−0.67	NS	21.89	< 0.05	0.50	0.79	−0.06
	Zebu cows	54	0.62	0.88	0.06	[0.76; 1]	−1.94	NS	14.24	< 0.05	0.44	0.72	−0.05
	Crossbred cows	16	0.75	0.88	0.12	[0.65; 1.12]	−0.98	NS	7.38	< 0.05	0.63	0.88	+0.01
Coarse fodder ingested (kg GM/d/cow)	All the cows	43	0.71	0.65	0.04	[0.58; 0.72]	−9.41	< 0.05	17.82	< 0.05	0.54	0.97	−0.15
	Zebu cows	37	0.86	0.69	0.04	[0.60; 0.77]	−7.29	< 0.05	15.99	< 0.05	0.34	0.47	+0.03
	Crossbred cows	6	0.31	0.40	0.30	[−1.20; 1]	−1.96	NS	1.33	NS	1.48	1.68	0.00
Feed concentrates ingested (kg GM/d/cow)	All the cows	78	0.95	0.97	0.01	[0.94; 0.99]	−2.34	< 0.05	65.17	< 0.05	0.49	0.82	−0.06
	Zebu cows	61	0.53	0.89	0.07	[0.76; 1.02]	−1.73	NS	13.37	< 0.05	0.39	0.61	−0.01
	Crossbred cows	17	0.83	0.88	0.05	[0.80; 0.97]	−2.61	< 0.05	19.86	< 0.05	0.72	1.23	−0.23
IDP intake from ingested ration (g IDP/d/cow)	All the cows	82	0.92	1.01	0.02	[0.97; 1.06]	0.56	NS	41.96	< 0.05	57.31	90.19	−3.73
	Zebu cows	65	0.60	0.94	0.07	[0.81; 1.07]	−0.87	NS	14.21	< 0.05	50.47	75.68	−0.33
	Crossbred cows	17	0.76	0.92	0.06	[0.81; 1.04]	−1.32	NS	15.59	< 0.05	74.91	128.62	−15.82
MFU intake from ingested ration (MFU/d/cow)	All the cows	82	0.93	1.02	0.02	[0.97; 1.06]	0.71	NS	44.07	< 0.05	0.52	0.79	−0.01
	Zebu cows	65	0.60	0.86	0.08	[0.71; 1.02]	−1.74	NS	10.91	< 0.05	0.40	0.55	+0.03
	Crossbred cows	17	0.58	0.80	0.14	[0.53; 1.07]	−1.46	NS	5.75	< 0.05	0.87	1.24	+0.09
Milk production (L/d/cow)	All the cows	82	0.82	0.85	0.02	[0.82; 0.89]	−8.40	< 0.05	48.37	< 0.05	0.88	1.69	−0.27
	Zebu cows	65	0.35	0.44	0.07	[0.30; 0.57]	−8.24	< 0.05	6.36	< 0.05	0.29	0.39	−0.04
	Crossbred cows	17	0.02	0.01	0.11	[−0.20; 0.23]	−9.07	< 0.05	0.12	NS	1.31	1.66	−0.18
Total cost of rations distributed (F CFA /d/cow)	All the cows	82	0.93	1.02	0.021	[0.98; 1.06]	0.80	NS	48.58	< 0.05	90.02	136.96	+8.43
	Zebu cows	65	0.65	0.72	0.06	[0.61; 0.83]	−5.06	< 0.05	13.09	< 0.05	68.13	94.83	−2.69
	Crossbred cows	17	0.81	1.01	0.09	[0.81; 1.20]	0.07	NS	10.15	< 0.05	147.34	200.80	+2.74
Gross profit margins (F CFA /d/cow)	All the cows	82	0.76	0.78	0.02	[0.73; 0.83]	−9.49	< 0.05	33.69	< 0.05	344.54	628.65	−99.98
	Zebu cows	65	0.28	0.40	0.08	[0.25; 0.55]	−7.63	< 0.05	5.08	< 0.05	138.40	189.82	−12.14
	Crossbred cows	17	0.06	0.12	0.12	[−0.11; 0.36]	−7.30	< 0.05	1.01	NS	586.54	675.93	0.00

kg GM= Kilogram of Gross Matter; d= day; MFU= Milk Forage Unit; IDP= Intestinal Digestible Proteins; 1 Euro= 655.957 FCFA; L= Litre; g= gram; For all variables presented in the table, the indicators derived from the robust linear regression analysis between predicted and actual values are reported; P < 0.05 indicates a significant difference; NS= Not significantly different (P > 0.05).

**Fig. 3:**
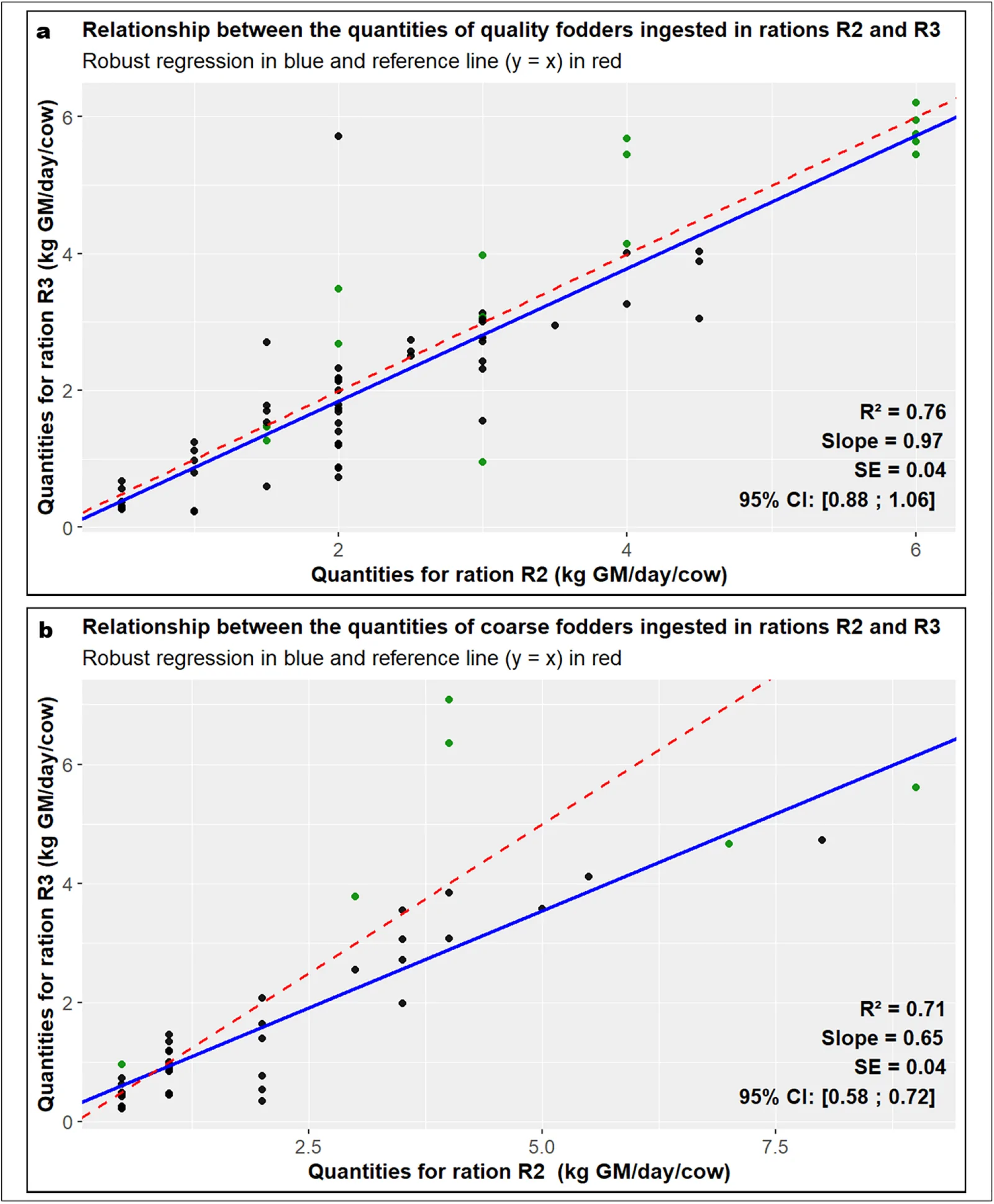
Correlations (R²) between R2 and R3 rations ingested for the quantity of quality fodder (kg GM/d/cow) and quantity of coarse fodder (kg GM/d/cow) variables.

**Fig. 4:**
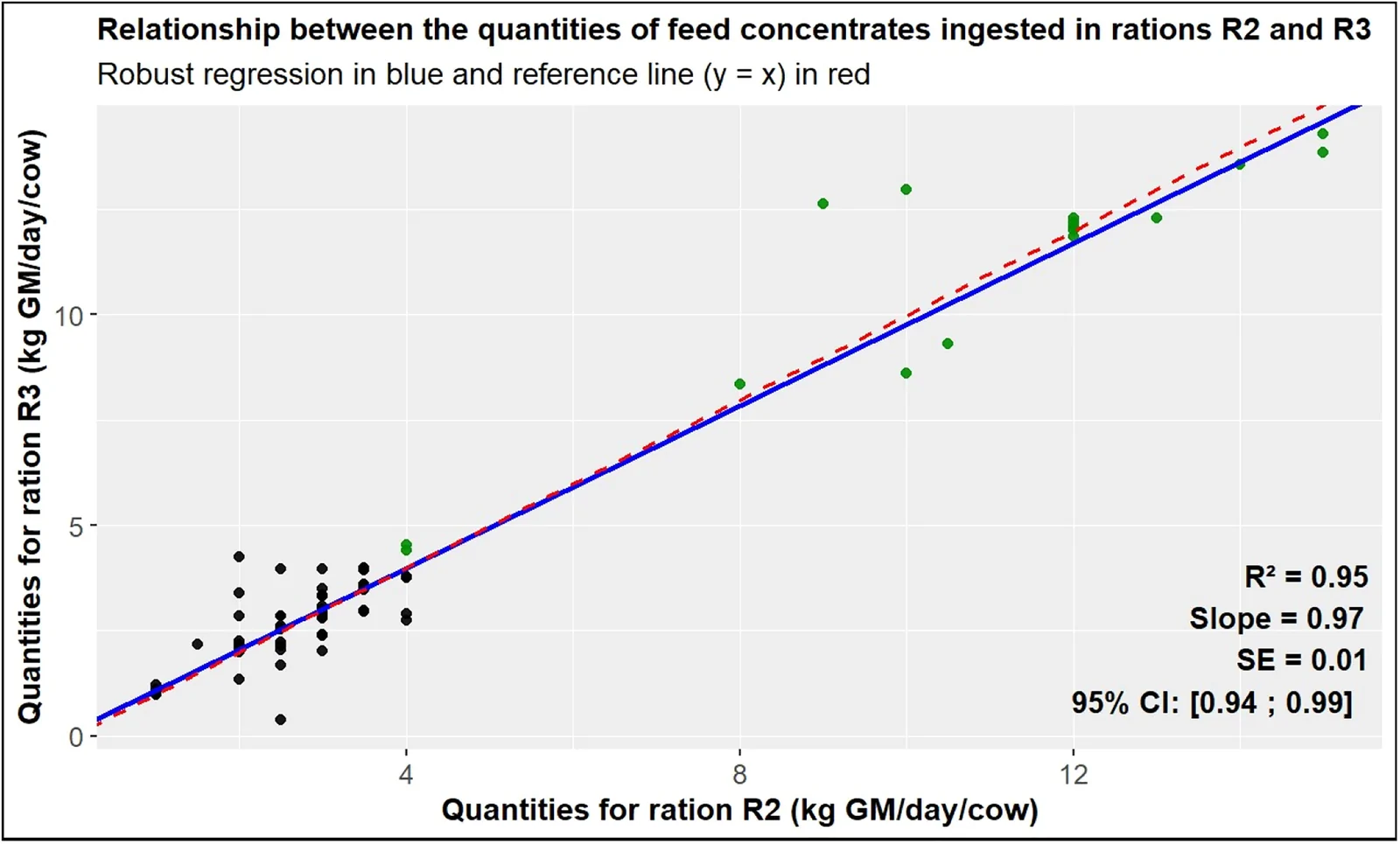
Correlations (R²) between the quantity of feed concentrates of R2 and R3 rations ingested (kg GM/d/cow).

The R² between feed intake quantities for R2 rations and those for R3 rations were 0.76 for quality fodder (Fig. 3a), 0.71 for coarse fodder (Fig. 3b), and 0.95 for feed concentrates (Fig. 4), respectively. With regard to prediction errors, the mean absolute errors (MAE) were 0.50 kg GM/d/cow for quality fodder, 0.54 kg GM/d/cow for coarse fodder, and 0.49 kg GM/d/cow for feed concentrates. The root mean square error (RMSE) values were slightly higher than the corresponding MAE values, with 0.79 kg GM/d/cow for quality fodder, 0.97 kg GM/d/cow for coarse fodder, and 0.82 kg GM/d/cow for feed concentrates. The mean bias error (MBE) values were close to zero, with −0.06 kg GM/d/cow for quality fodder, −0.15 kg GM/d/cow for coarse fodder, and −0.06 kg GM/d/cow for feed concentrates (Table 6).

The correlations between R2 and R3 rations ingested varied according to cow type. In terms of quality fodder, the R² values were 0.62 and 0.75 for zebu and crossbred cows, respectively, with MBE values of −0.05 and +0.01 kg GM/d/cow. With regard to coarse fodder, the R² values were 0.86 and 0.31 for zebu and crossbred cows, respectively, and the MBE values were 0.03 and 0.00 kg GM/d/cow. In terms of feed concentrates, the R² values were 0.53 and 0.83 for zebu and crossbred cows, respectively, with MBE values of −0.01 and −0.23 kg GM/d/cow (Table 6).

R2= final ration recommended to dairy farmers (predicted ration); R3= ration actually implemented by dairy farmers (actual ration); kg GM= Kilogram of Gross Matter; Black dots= zebu cows; Green dots= crossbred cows; SE= standard error of the slope; 95% CI= slope confidence interval.

Quantity of quality fodder ingested (Mean absolute error= 0.50 kg GM/d/cow; Root mean square error= 0.79 kg GM/d/cow; Mean bias error= −0.06 kg GM/d/cow); Quantity of coarse fodder ingested (Mean absolute error= 0.54 kg GM/d/cow; Root mean square error= 0.97 kg GM/d/cow; Mean bias error= −0.15 kg GM/d/cow).

R2= final ration recommended to dairy farmers (predicted ration); R3= ration actually implemented by dairy farmers (actual ration); kg GM= Kilogram of Gross Matter; Black dots= zebu cows; Green dots= crossbred cows; SE= standard error of the slope; 95% CI= slope confidence interval; (Mean absolute error= 0.49 kg GM/d/cow; Root mean square error= 0.82 kg GM/d/cow; Mean bias error= −0.06 kg GM/d/cow).

**Fig. 5:**
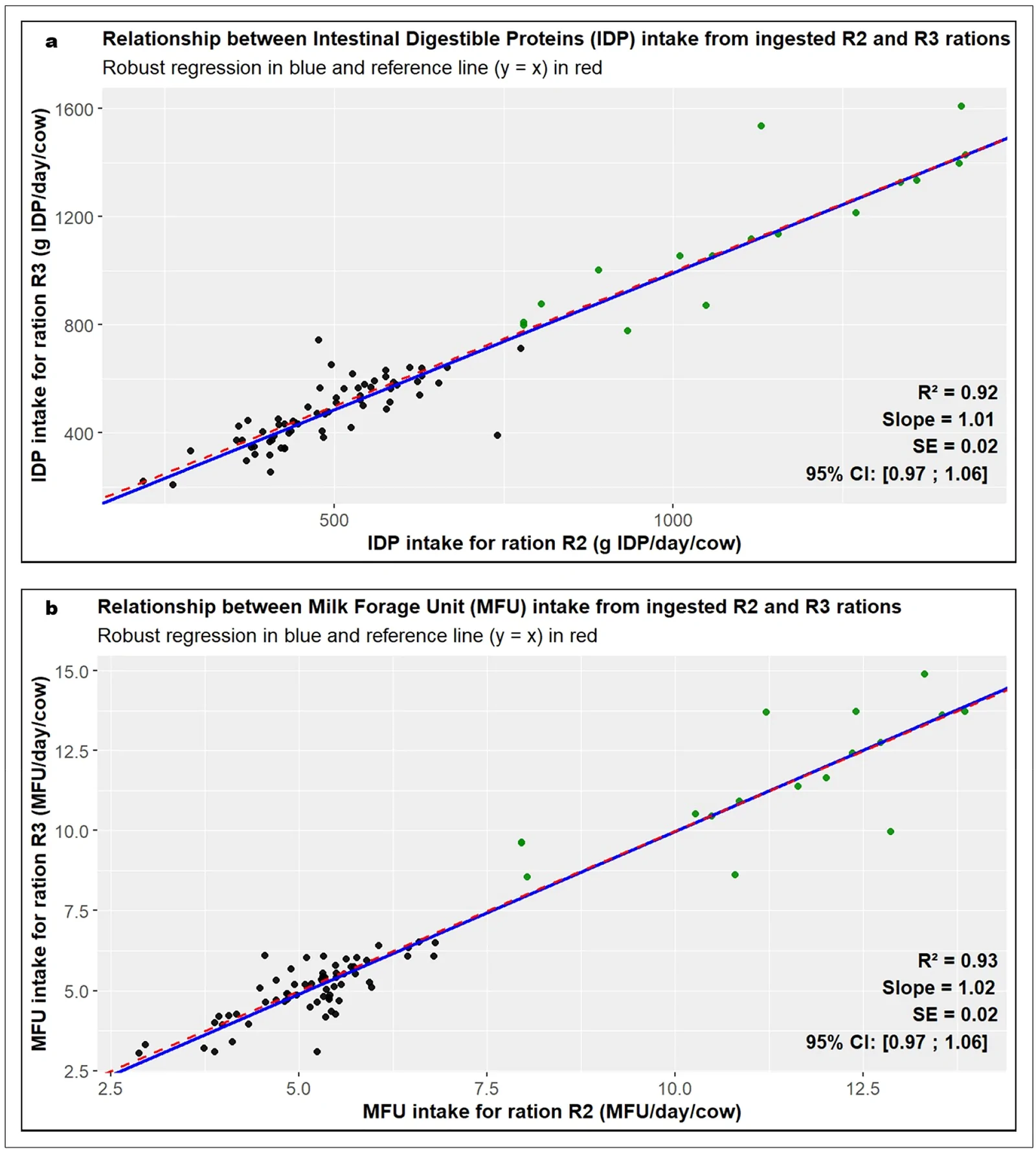
Correlations (R²) between: IDP (g IDP/d/cow) and MFU (MFU/d/cow) intake from ingested R2 and R3 rations.

With regard to IDP and MFU intake, the R²s between R2 and R3 rations were 0.92 and 0.93, respectively (Fig. 5a and b). With regard to prediction errors, the MAE were 57.31 *g* IDP/d/cow for IDP and 0.52 MFU/d/cow for MFU. The RMSE values were slightly higher than the corresponding MAE values, with 90.19 *g* IDP/d/cow for IDP and 0.79 MFU/d/cow for MFU. The MBE value was somewhat further from zero for IDP (−3.73 *g* IDP/d/cow) and close to zero for MFU (−0.01 MFU/d/cow).

The correlations between R2 and R3 rations ingested were close across cow type. In terms of IDP, the R² values were 0.60 and 0.76 for zebu and crossbred cows, respectively, with MBE values of −0.33 and −15.82 g IDP/d/cow. With regard to MFU, the R² values were 0.60 and 0.58 for zebu and crossbred cows, respectively, with MBE values of +0.03 and +0.09 MFU/d/cow (Table 6).

R2= final ration recommended to dairy farmers (predicted ration); R3= ration actually implemented by dairy farmers (actual ration); MFU= Milk Forage Unit; IDP= Intestinal Digestible Proteins; Black dots= zebu cows; Green dots= crossbred cows; SE= standard error of the slope; 95% CI= slope confidence interval.

[IDP intake from ingested ration= ∑ Ingested quantity of each feed in the ration **×** feed DM coefficient **×** feed IDP value (Mean absolute error= 57.31 *g* IDP/d/cow; Root mean square error= 90.19 g IDP/d/cow; Mean bias error= −3.73 *g* IDP/d/cow)]; [MFU intake from ingested ration= ∑ Ingested quantity of each feed in the ration **×** feed DM coefficient **×** feed MFU value (Mean absolute error= 0.52 MFU/d/cow; Root mean square error= 0.79 MFU/d/cow; Mean bias error= −0,01MFU/d/cow)]

**Fig. 6:**
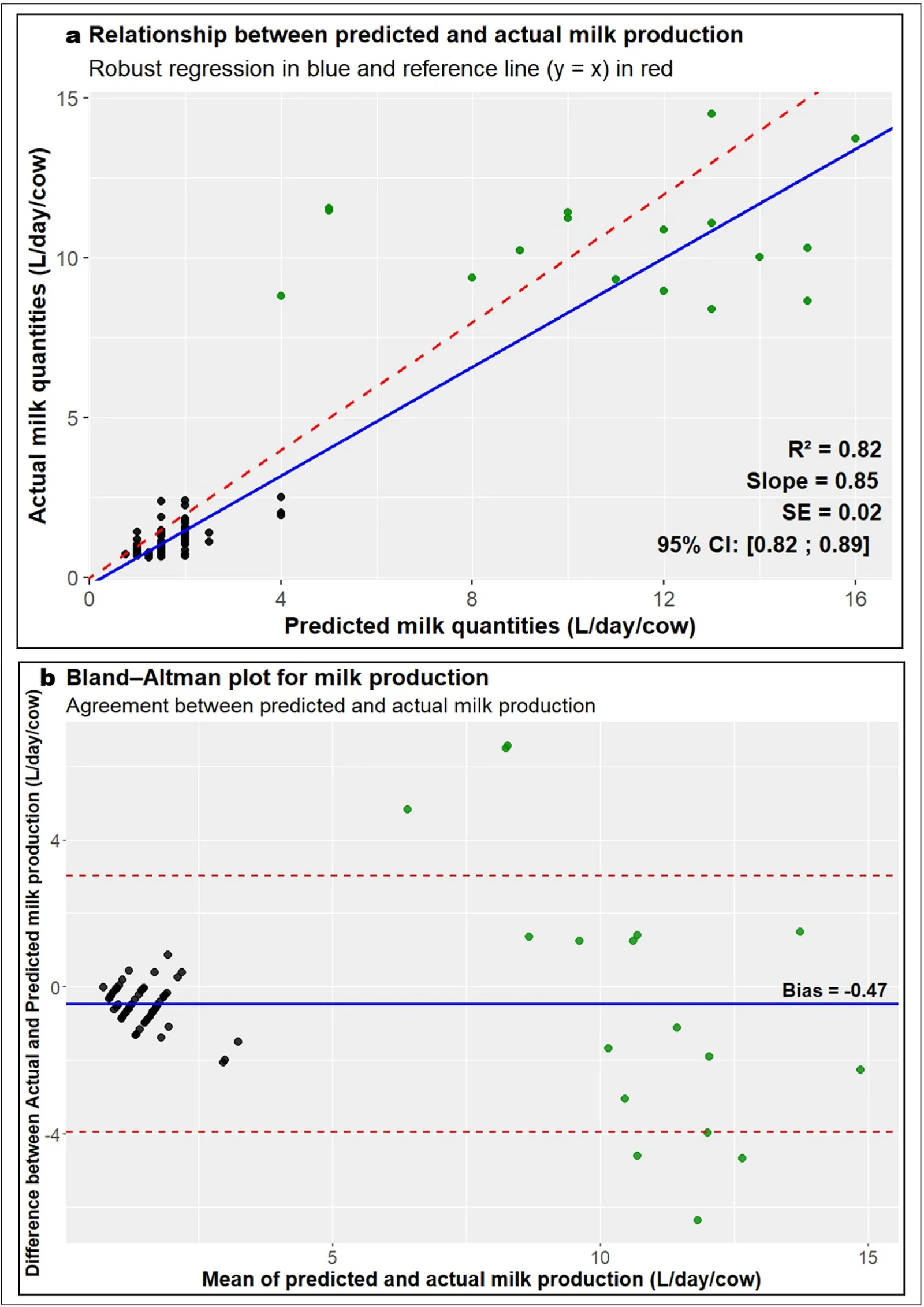
Correlations (R²) and Bland-Altman plot between predicted and actual milk production (L/d/cow).

In terms of milk production, the R² between predicted and actual milk production was 0.82 (Fig. 6a). With regard to prediction errors, the mean absolute error (MAE), root mean square error (RMSE), and mean bias error (MBE) were 0.88, 1.69, and −0.27 L/d/cow, respectively (Table 6).

The Bland-Altman analysis showed that most observations fell within the 95% limits of agreement (−3.96 to 3.02 L/d/cow), with a mean bias of −0.47 L/d/cow (Fig. 6b).

The correlations between predicted and actual milk production varied according to cow type. Among zebu cows, the R² was 0.35, with MAE, RMSE, and MBE values of 0.29, 0.39, and −0.04 L/d/cow, respectively. In contrast, the correlation was much weaker for crossbred cows (R²= 0.02), with MAE, RMSE, and MBE values of 1.31, 1.66, and −0.18 L/d/cow, respectively (Table 6).

Predicted milk production= dairy farmer's milk production target per cow corresponding to the quantity to be milked; Actual milk production= average production over the last 7 days of the trial, corresponding to the quantity milked by the herdsman; L= litre; Black dots= zebu cows; Green dots= crossbred cows; SE= standard error of the slope; 95% CI= slope confidence interval; (Mean absolute error= 0.88 L/d/cow; Root mean square error= 1.69 L/d/cow; Mean bias error= −0.27 L/d/cow)

**Fig. 7:**
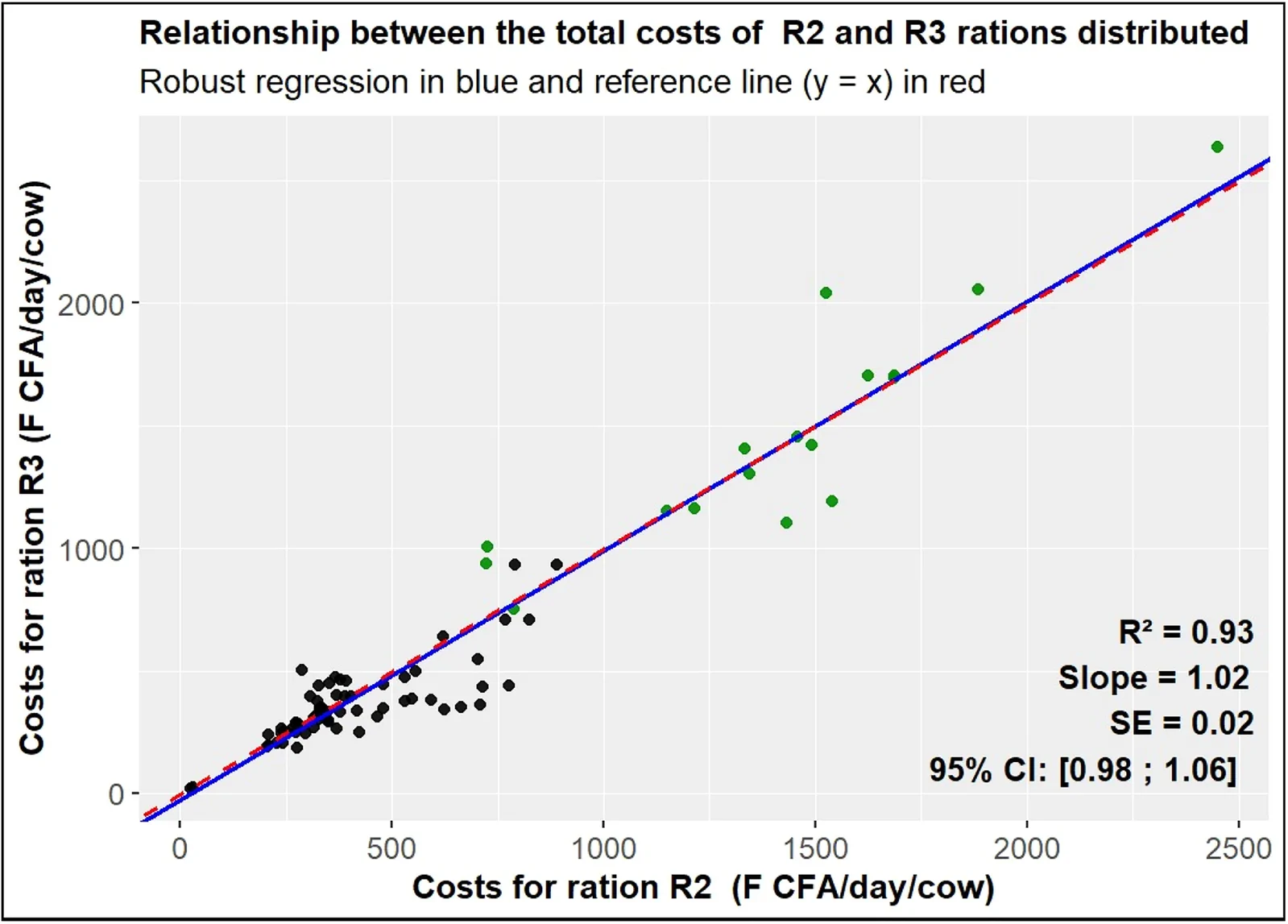
Correlations (R²) between total cost of R2 and R3 rations distributed (F CFA/d/cow).

In terms of the total cost ration, the R² between R2 and R3 rations distributed was 0.93 (Fig. 7). With regard to prediction errors, the MAE, RMSE, and MBE were 90.02, 136.96 and +8.43 F CFA /d/cow, respectively (Table 6).

The correlation between R2 and R3 rations distributed varied according to cow type. Among zebu cows, the R² was 0.65, with MAE, RMSE, and MBE values of 68.13, 94.83, and −2.69 F CFA /d/cow, respectively. Among crossbred cows, the R² was 0.81, with MAE, RMSE, and MBE values of 147.34, 200.80, and +2.74 F CFA /d/cow, respectively (Table 6).

R2= final ration recommended to dairy farmers (predicted ration); R3= ration actually implemented by dairy farmers (actual ration); Black dots= zebu cows; Green dots= crossbred cows; SE= standard error of the slope; 95% CI= slope confidence interval; Total cost of rations distributed= estimated ration cost based exclusively on feed prices and distributed quantities (Mean absolute error= 90.02 F CFA /d/cow; Root mean square error= 136.96 F CFA /d/cow; Mean bias error= −8.43 F CFA/d/cow); 1 Euro= 655.957 F CFA.

**Fig. 8:**
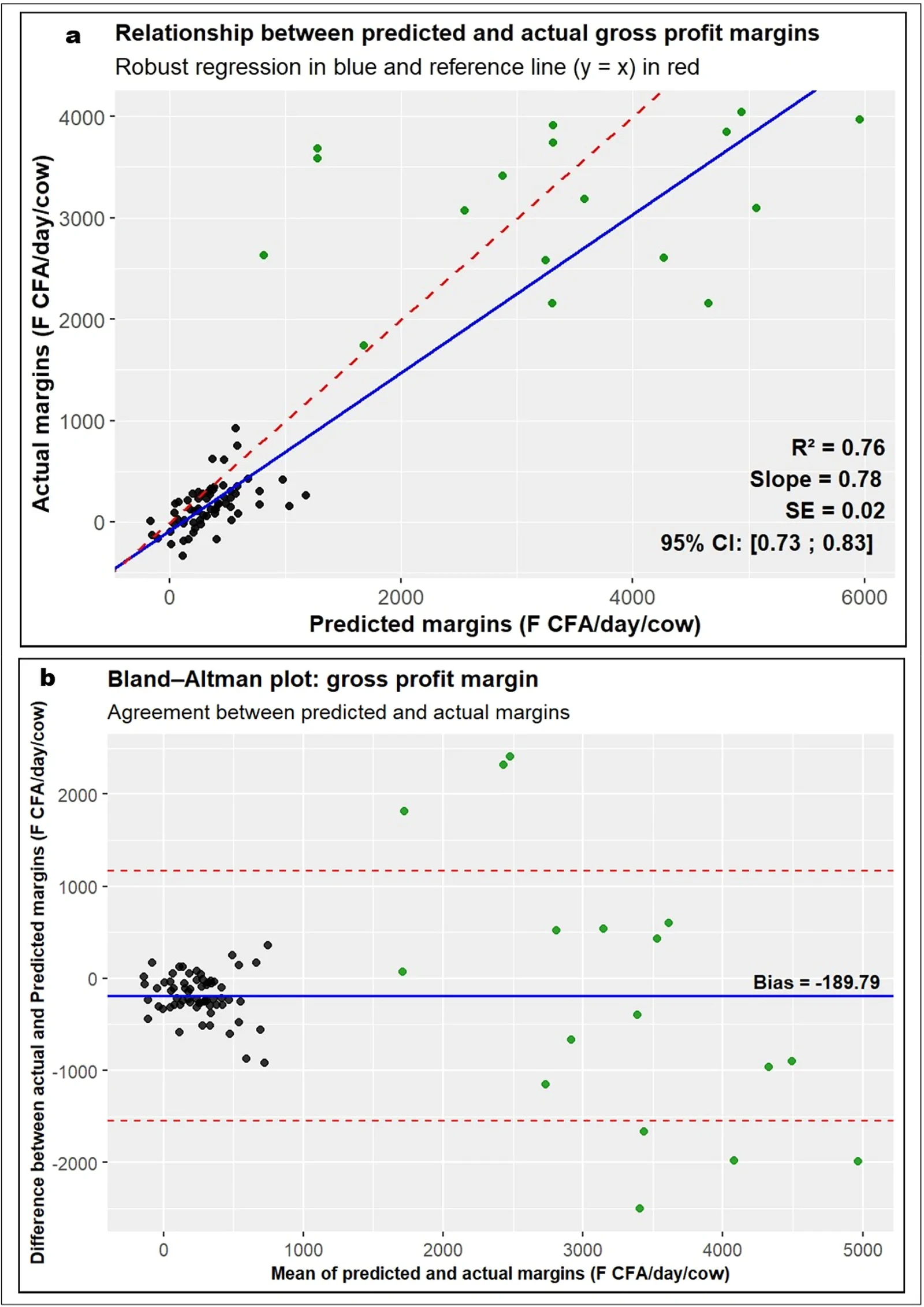
Correlations (R²) and Bland-Altman plot between predicted and actual gross profit margin (F CFA/d/cow).

In terms of gross profit margin, the R² between predicted and actual gross profit margin was 0.76 (Fig. 8a). With regard to prediction errors, the MAE, RMSE, and MBE were 344.54, 628.65, and −99.98 F CFA/d/cow, respectively (Table 6).

The Bland-Altman analysis showed that most observations fell within the 95% limits of agreement (−1550 to 1171 F CFA/d/cow), with a mean bias of −190 F CFA/d/cow (Fig. 8b).

The correlations between predicted and actual gross profit margin varied according to cow type. Among zebu cows, the R² was 0.28, with MAE, RMSE, and MBE values of 138.40, 189.82, and −12.14 F CFA/d/cow, respectively. In contrast, the correlation was much weaker for crossbred cows (R²= 0.06), with MAE, RMSE, and MBE values of 586.54, 675.93, and 0.00 F CFA/d/cow, respectively (Table 6).

R2= final ration recommended to dairy farmers (predicted ration); R3= ration actually implemented by dairy farmers (actual ration); Gross profit margins= gross margin estimated considering only milk sales revenue and feed costs; 1 Euro= 655.957 F CFA; Black dots= zebu cows; Green dots= crossbred cows; SE= standard error of the slope; 95% CI= slope confidence interval; (Mean absolute error= 344.54 F CFA /d/cow; Root mean square error= 628.65 F CFA /d/cow; Mean bias error= −99.98 F CFA/d/cow).

### 3.3 Dairy farmers’ satisfaction with the use of *Jaɓnde*

Dairy farmers stated that they were satisfied with the actual milk production from 82% of crossbred cows and 86% of zebu cows. In their view, actual milk production levels for all crossbred cows and 99% of zebu cows would not have been achieved without the help of *Jaɓnde*. For crossbreds, they reported that milk production had increased significantly in 35% of cases, increased slightly in 53%, remained stable in 6%, and dropped slightly in 6% of cases. Among zebu cows, dairy farmers reported that milk production had risen sharply in 52% of cases, increased slightly in 43%, and remained stable in 5% of cases.

Dairy farmers expressed satisfaction with gross profit margins achieved for 82% of crossbred cows and 77% of zebu cows.

**Fig. 9:**
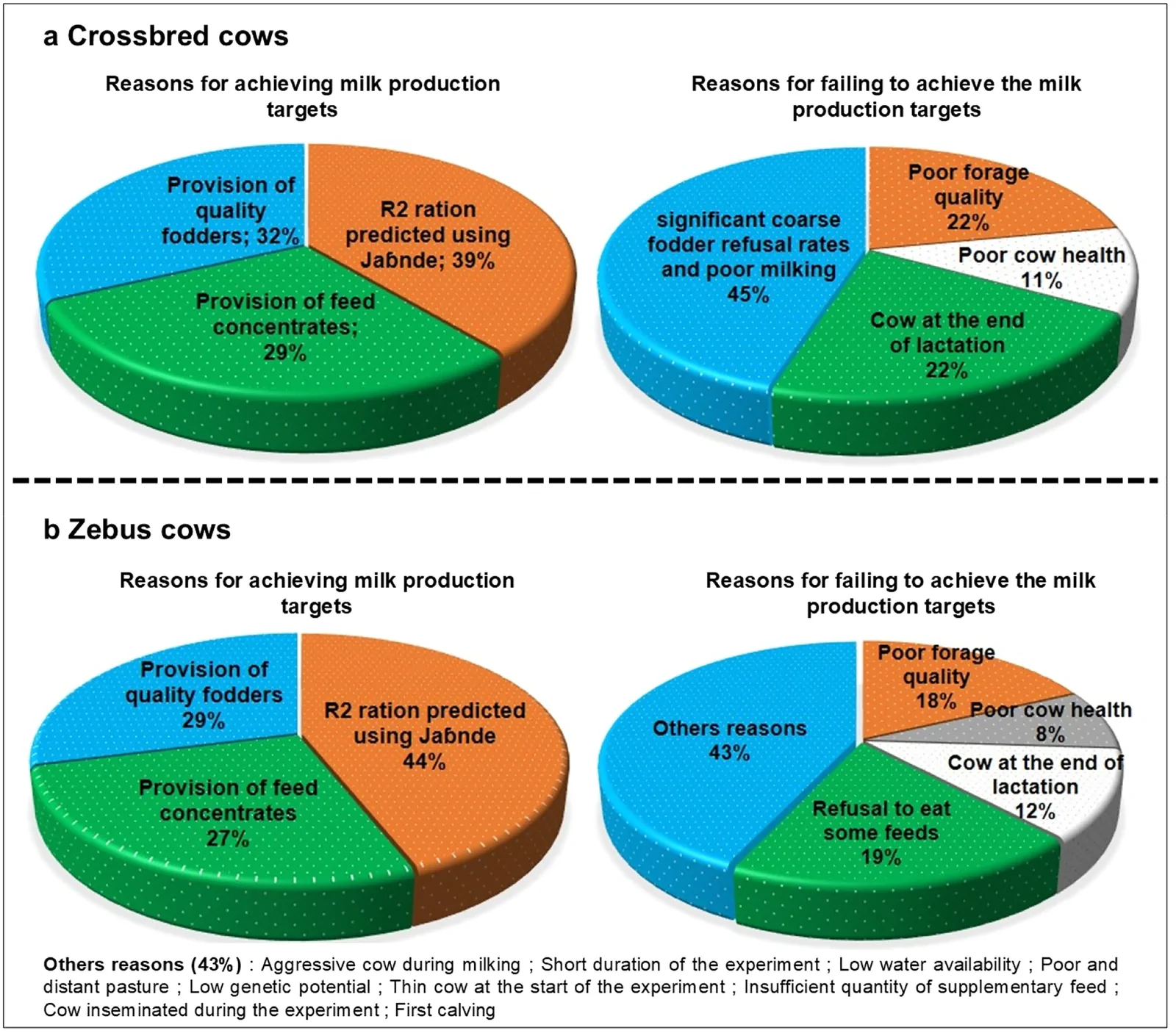
Reasons for achieving or failing to achieve milk production targets according to dairy farmers.

Reasons given by dairy farmers for achieving milk production targets (predicted milk production) were as follows: (i) R2 ration predicted using *Jaɓnde*; (ii) provision of quality fodder, and (iii) provision of feed concentrates. As for the reasons given by some farmers for failing to achieve milk production targets, the following were mentioned: (i) significant coarse fodder refusal rates; (ii) poor milking by the herdsman; (iii) poor forage quality; (iv) poor cow health; (v) cow's lactation stage (end of lactation); and (v) other reasons (Fig. 9a and b). Percentages corresponding to each modality, calculated by cow type, were standardised on a 0 −100% scale.

The majority of dairy farmers (93%) stated that actual milk production from cows rationed with the *Jaɓnde* tool was higher than from cows that had not been rationed using the tool. They also felt that rationing with *Jaɓnde* had raised their awareness on several levels. Lastly, dairy farmers said that *Jaɓnde* was an effective tool for improving both cows' milk production and their income (Table 7).

**Table 7:** Dairy farmers' perception of the impact of cow rationing with *Jaɓnde*.

Variables	Modalities	Frequency (%)
Milk production differences between rationed and non-rationed cows	The quantity of milk produced by rationed cows is greater than that of non-rationed cows using the *Jaɓnde* tool	93
	The quantity of milk produced by rationed cows is lower than that of non-rationed cows using the *Jaɓnde* tool	5
	Milk quantities from cows rationed and not rationed with the *Jaɓnde* tool are similar	2
Impact of the trial on cow feeding practices	Improved estimate of feed quantities to be distributed	98
	Better understanding of the choice of feed to be distributed to improve production	90
	A clearer picture of the expenditure incurred	75
	Better understanding of the importance of good fodder storage as part of feed management	73
Use of *Jaɓnde* as a lever for improving cow milk production and income	Very good tool	95
	Good tool	5

For variables “Milk production differences between rationed and non-rationed cows” and “Use of *Jaɓnde* as a lever for improving cow milk production and income” recorded at the dairy farmer level, percentages were calculated based on the total number of surveyed dairy farmers. For the variable “impact of the trial on cow feeding practices”, recorded at the dairy farmer level, percentages were calculated for each modality based on the number of dairy farmers who selected it.

## 4 Discussion

The study aimed to: (i) characterise and compare the technical and economic performance of the two main cow types (zebu and crossbred) under dairy farmer-implemented rations (R3 ration); (ii) assess *Jaɓnde* along two complementary dimensions: the fidelity with which dairy farmers implemented the predicted rations (R2 ration), and the predictive performance of the tool for feed intake, milk production, ration cost, and gross profit margin; and (iii) assess dairy farmers’ satisfaction with the use of *Jaɓnde* for managing the feeding of their dairy cows.

### 4.1 Zebu and crossbred cow feed and milk performance

Feed intake by crossbred cows averaging 5 years of age and 434 kg live weight was higher than that of zebu cows averaging 7 years of age and 248 kg live weight. This is due to the fact that crossbred cows were being fed at the trough, with an average grazing time of 3 h/d. For zebu cows, however, the feeding system was based on pasture grazing (9 h/d), with supplementary feeding at the trough. Feeding practices for zebu cows are very similar to those described by Sodré et al. (2022) on extensive dairy farms in Western Burkina Faso. In such systems, dairy cows were fed on pasture (8 to 21 h/d) and were supplemented during the dry season with quality and coarse fodder (1 to 9 kg DM/d/cow) and feed concentrates (0.2 to 1.3 kg GM/d/cow). As for crossbred cows, their feeding system is similar to that of market-oriented dairy farmers from the Kadigo, Houet, and Comoé provinces (Vall et al., 2021). These farmers' dairy cows were mainly Sudanese Fulani zebu cows crossed with exotic dairy breeds (Holstein, Brune des Alpes). These cows were less dependent on grazing, with a pasture intake of 1.3 kg/d/TLU. Fodder and feed concentrate intake were 5.6 and 5.7 kg GM/d/TLU, respectively.

The large amounts of fodder and feed concentrates provided to crossbred cows enabled them to produce greater quantities of milk (10.6 L/d/cow) than zebu cows (1.2 L/d/cow) at lower production costs. Such high productivity of crossbred cows compared to zebu cows was reported by Kassa et al. (2016a) in West Africa. Ouédraogo et al. (2023) also noted that dairy farm production levels were influenced in particular by the presence of crossbred cows and exotic breeds. This is because zebu cows are not, strictly speaking, dairy breeds; their genetic potential for milk production is therefore lower than that of crossbreds, which are better suited to milk production. In addition, longer grazing sessions did not improve zebu cow performance. According to the authors (Molina-Flores et al., 2020; Zampaligré et al., 2013), although natural pasture provides free forage resources, it has declined in both quantitative and qualitative terms as a result of drought, desertification, and the expansion of farmland.

Milk yields recorded in this study are consistent with those reported in previous studies. Ouattara et al. (2025) noted an increase in milk production from 1.1 to 1.3 L/d/cow for zebu cows and from 6.4 to 11.7 L/d/cow for crossbred cows following the introduction of agroecological technologies for fodder diversification and co-product recycling on dairy farms. Depending on the feeding system, Sodré et al. (2022) recorded a production of around 1 L/d/cow with zebu cows. On farms with a large proportion of crossbred cows, Vall et al. (2021) and Sib et al. (2018) recorded yields of 7 and 13 L/d/cow, respectively. In peri‑urban livestock farms of Fada N'Gourma, where 98.6% of the animals are local breeds, yields of 1.95 L/d/cow were reported by Ouédraogo et al. (2023). In Benin, significant differences have been recorded between different cattle breeds: Girolando cows (4.7 L/d), Borgou cows (0.8 L/d/cow), Azawak × Lagunaire crossbreds (0.7 L/d/cow), and Lagunaire cows (0.4 L/d/cow) (Kassa et al., 2016b). Zebu cows' milk production could have been further improved with multiple daily milkings. Sissao et al. (2018) did indeed record yields of 3.1 L/d/cow following a switch from three to two milkings and 1.98 L/d/cow for two milkings in Gobra zebu cows.

The found milk production levels may also be explained in terms of nutrient utilisation mechanisms among dairy cows. Indeed, a better match between nutrient supply and dairy cows' requirements is likely to improve nitrogen use efficiency by reducing losses and increasing the availability of amino acids for milk synthesis. Furthermore, ration optimisation may modify rumen fermentation, leading to improved protein digestion and a greater supply of absorbable nutrients at the intestinal level. These adjustments also influence nutrient partitioning between maintenance and production functions, thereby promoting milk production (Castillo et al., 2025a, 2025b; Castillo et al., 2000; Jami et al., 2014; Lucy et al., 2013). Although these mechanisms were not directly measured in the present study, they offer a plausible physiological framework for interpreting the production responses found with *Jaɓnde*-optimised rations, in which a closer match between nutrient supply and requirements is expected to enhance the efficiency with which ingested nutrients are converted into milk.

It is clear that crossbred cows offer significantly greater milk performance than zebu cows. They also generated substantially higher gross profit margins (3142 ± 720 vs 158 ± 224 F CFA/d/cow). These results suggest that crossbred cows could play an important role in improving milk production and farm profitability, thereby contributing to achieving self-sufficiency in milk production. However, this improvement in milk production and profitability raises questions regarding its genetic, environmental, and economic implications, as well as its sustainability. In this study, the assessment of milk production costs did not factor in other farm costs (depreciation, veterinary care, labour), aside from those related to rations. In addition, artificial insemination and crossbreeding are not rigorously used in West Africa. Crossbreeding is very often haphazard and does not follow any logic (Kassa et al., 2016a). Additionally, the animals resulting from these genetic improvements may be less resilient to local weather conditions and diseases and ill-suited to forage resources (Kassa et al., 2016a). Vall et al. (2021) had expressed concern about genetic improvement practices and excessive use of feed concentrates on the most intensive farms in West Africa. According to Nsangou et al. (2021), the mortality rate is three times higher in crossbreds than in zebus between zero and 36 months.

Furthermore, although crossbred cows produced higher milk yields than zebu cows, *Jaɓnde*'s relative impact on improving milk yields was more pronounced among zebu cows. On average, milk production rose by 33% in zebu cows, versus 8% in crossbreds. In addition, dairy farmers stated that 52% of zebu cows had achieved a significant increase in milk production, versus 35% of crossbred cows. These results suggest that the *Jaɓnde* tool seems particularly well suited to support milk production in zebu cows. However, the higher initial milk production of crossbred cows compared with zebu cows (9.8 vs 0.9 L/d/cow) may have influenced the scope for improvement found between the two cow types, as the potential for relative increases in milk production is not necessarily the same at different baseline production levels.

Beyond its technical and economic functions, *Jaɓnde* also provides a ration-based estimate of enteric methane (CH₄) production. As for milk production and gross margin, this output is a model prediction rather than a direct measurement: it is derived from the quantity and composition of the ingested ration and from the fermentation of organic matter that follows in the rumen. In the present trial, the predicted potential CH₄ production differed between cow types, being higher in crossbred cows (36 ± 7 g/d/cow) than in zebu cows (20 ± 3 g/d/cow), in line with their greater feed intake and higher milk production. Because the estimate responds directly to the formulated ration, it allows the tool to flag, at the formulation stage, rations associated with higher predicted methane output, and thus to favour, among nutritionally and economically equivalent options, those that limit enteric emissions. This positions *Jaɓnde* as a decision-support tool acting simultaneously on three levels: technical, through the formulation of balanced rations; economic, by steering farmers towards least-cost rations; and environmental, by drawing attention to rations that reduce predicted enteric methane intensity. These estimates should nonetheless be interpreted with caution, as they were not validated against direct CH₄ measurements; their experimental verification under field conditions constitutes a relevant avenue for future work.

### 4.2 Implementation of R3 ration close to R2 ration and reasonable predictive performance of *Jaɓnde*

Overall, the dairy farmers succeeded in implementing actual rations (R3) that were close to the predictive rations (R2), despite using local units of measurement (box, rope, plastic bag, hollow container) that differ from *Jaɓnde's* (kg).

With regard to fodder quantities, a slight under-ingestion was found among zebu cows compared with the R2 ration, with mean differences of −0.19 and −0.45 kg GM/d/cow for quality fodder and coarse fodder, respectively. Conversely, a slight over-ingestion was found among crossbred cows (+0.19 and +0.15 kg GM/d/cow for quality fodder and coarse fodder, respectively). For feed concentrates, the overall mean difference, for both cow types was small (+0.04 kg GM/d/cow), indicating close proximity between R2 and R3 rations.

Because a high R² reflects the existence of an association rather than predictive agreement as such, these interpretations below rest primarily on the slope-against-unity test (Table 6) and on the prediction-error metrics.

Associations between the ingested R2 and R3 rations were strong for most variables (R² > 0.70), including the cost of the rations distributed (R²= 0.93), indicating a generally good overall agreement between R2 and R3 rations in both nutritional and economic terms. This pattern was not uniform across all variables. Indeed, for coarse fodder, the slope was clearly below unity (β1= 0.65 for all cows; 95% CI= [0.58; 0.72]), reflecting the higher refusal rate found for this feed. For quality fodder, feed concentrates, and IDP and MFU intake, the slopes were close to, or statistically compatible with, unity. The low mean absolute error (MAE ≤ 0.54 kg GM/d/cow) and root mean square error (RMSE ≤ 0.97 kg GM/d/cow), together with the slightly negative mean bias error (MBE ≤ −0.15 kg GM/d/cow), indicate good accuracy and only a moderate tendency of the tool to overestimate predicted quantities. The found discrepancies could be explained by the feeding behaviour of the dairy cows, variability in forage quality, the use of local measurement units, and feed-stock management strategies leading dairy farmers to adjust distributed quantities according to resource availability.

With regard to cow type, correlations varied, particularly for coarse fodder. The association between R2 and R3 ration was higher among zebu cows (R²= 0.86) than for crossbred cows (R²= 0.31). This difference suggests greater individual variability in ration implementation among crossbred cows. However, it should be noted that these results are based on unequal sample sizes, comprising 37 zebu cows and 6 crossbred cows, which may limit the stability of the estimates in the latter group. Despite this variability, MBE values remained very low in both groups (+0.03 and +0.00 kg GM/d/cow, respectively, for zebu and crossbred cows), indicating the absence of substantial systematic bias.

The observation of R3 rations being higher than R2 rations in some cows (Fig. 3) is not surprising in the context of the study area. Dairy farmers very often associate an increase in the quantities of feed distributed with an increase in milk production, which can lead to the over-distribution of certain feeds. However, the use of *Jaɓnde* appears to have contributed to strengthening their knowledge of ration formulation. The farmers notably reported that the trial had enabled them to better estimate the quantities of feed to be distributed and to better understand the choice of feeds according to the cows’ requirements and production objectives. These results are consistent with those of Vall et al. (2021) in Burkina Faso, who observed a tendency towards over-distribution of feeds in market-oriented dairy farms, this practice being perceived as a simple means of increasing milk production. Such a practice may, however, reduce feed efficiency and increase the risk of nutritional imbalances, particularly acidosis.

In terms of production performance, the strength of the predicted-actual association varied markedly across variables and cow types and was therefore not interpreted as direct evidence of predictive accuracy. For all cows combined, the association was moderate to strong for milk production (R²= 0.82) and gross profit margin (R²= 0.76). But the regression slopes were significantly lower than unity (β1= 0.85 and 0.78, respectively; p < 0.05), indicating a proportional tendency to underestimate the highest values rather than perfect agreement.

The association was, moreover, much weaker in crossbred cows (R²= 0.02 for milk production; 0.06 for gross profit margin) than in zebu cows (R²= 0.35 and 0.28), so that the predictive performance for this subgroup should be regarded as inconclusive, owing partly to its small size (n = 17 crossbred cows).

The low MAE (0.88 L/d/cow) and RMSE (1.69 L/d/cow) values indicate reasonably good prediction for milk production. For gross profit margin, although absolute errors were higher (MAE= 344.54 F CFA/d/cow; RMSE= 628.65 F CFA/d/cow) owing to the scale of the variable, the results remained consistent with the actual trends. The negative MBE values for milk production (−0.27 L/d/cow) and gross profit margin (−99.98 F CFA/d/cow) indicate a slight overestimation by the tool (MBE defined as actual minus predicted).

Bland-Altman analyses generally confirmed these results, with most observations lying within the 95% limits of agreement. The mean biases found for milk production (−0.47 L/d/cow) and gross profit margin (−190 F CFA/d/cow) remained low, suggesting a good level of agreement between predicted and actual values. Overall, these results indicate that *Jaɓnde* predictions are generally reasonable and consistent with actual performance under real farming conditions.

Most of the correlations found in this study are close to those of Da Silva and Costa (2025), who reported an R^2^ of 0.91 between weight predicted using *CalfSim* (a tool for dairy calf ration optimisation) and actual weight. Like *CalfSim, Jaɓnde* does not include farm-specific variables (such as disease outbreaks, environmental stress, and herdsman behaviour) that may have influenced the tool's predictions.

In addition, these results show that with a suitable rationing tool, dairy farmers are able to adopt an individual approach to ration formulation, in accordance with zootechnical principles. This qualifies the findings of Darré et al. (2007), who reported that farmers and animal scientists draw on different modes of reasoning in their approach to livestock farming practices. According to Richard et al. (2019), most farmers use supplements on an animal batch basis (draught cattle, young cattle (1 to 3 years old), lactating cows). The technical support provided with *Jaɓnde* and the follow-up activities carried out with farmers in our study played a key role in the shift towards individualised rations. This interpretation is consistent with Le Gal et al. (2011), who found that the use of simulation tools (*Dalib* and *Cikeda*) on dairy and polyculture/livestock farms not only helped with decision-making, but also gradually enhanced farmers' agronomic and zootechnical knowledge.

Overall, dairy farmers reported being satisfied with the performance achieved using the rations formulated with the *Jaɓnde* tool. >90% of them reported a perceived increase in milk production. Regarding the gross profit margin, dairy farmers reported being satisfied with the results obtained in 82% of crossbred cows and 77% of zebu cows. These levels of satisfaction were associated with a very positive perception of *Jaɓnde*, with dairy farmers considering it a useful tool for improving milk production and income in smallholder dairy farms. These findings suggest that a digital ration formulation tool designed in line with local conditions may promote the adoption of digital decision-support tools in smallholder dairy farms in Burkina Faso and, more broadly, in similar contexts across sub-Saharan Africa. In Italy, many dairy farms are already highly digitalised and rely on farm management software, automated feeding systems and connected sensors. Although these technologies are perceived as effective tools for supporting day-to-day management, their uptake remains constrained by high investment costs and limited interoperability between different systems (Lamanna et al., 2026). The development of digital decision-support tools adapted to local conditions, combined with improved economic accessibility and greater interoperability, could therefore facilitate their wider adoption in dairy farming systems.

### 4.3 Study limitations and strengths

The limitations of this study can be divided into two main groups: those that are inherent to the *Jaɓnde* tool, and those that are unrelated to it, such as those linked to the trial protocol and the livestock farming context.

The *Jaɓnde* tool does not take feed refusals into account when formulating rations. The rations developed by the tool (R2 rations) are therefore assumed to be fully ingested by the cows. Yet the results of the trial revealed high refusal rates (Table 4). Failure to account for refusals thus limits the tool's ability to fine-tune rations to the actual needs of the cows.

Additionally, the version of *Jaɓnde* used (Excel prototype) is not particularly convenient. It requires a laptop with a decent battery life for ration co-design sessions with dairy farmers and on their farms. To address this limitation, a 1.0 version of the tool has been developed and is available free of charge on Android mobiles via Google Play Store (not yet available on Apple Store), as well as on the Internet (https://jabnde.cirad.fr/). Pilot tests for this version of *Jaɓnde* are currently underway in Senegal and North Cameroon. However, the current versions of *Jaɓnde* are only available in French, which may limit their accessibility among non-French-speaking users and restrict their dissemination in some sub-Saharan African contexts. Developing multilingual versions adapted to local linguistic contexts would be an important step towards facilitating wider adoption of the tool.

Finally, a technical adviser is required when using *Jaɓnde*, potentially generating costs for dairy farmers and limiting the frequency with which rations can be adjusted in response to changes in lactation stage or fodder availability. However, any literate dairy farmer with prior training could use the mobile version of the tool on their own.

A promising avenue for improvement would be to integrate species- and fodder-specific refusal coefficients directly into the optimisation process, so that the tool formulates rations on the basis of expected distributed quantities rather than ingested quantities. Such an adjustment would increase the biological realism of the predicted rations and, in turn, the predictive performance of *Jaɓnde*, particularly for coarse fodder, for which the highest refusal rate was found.

During the trial, the lack of protocol for health prophylaxis may have influenced cows' intake. The nutritional values of all feed resources used were not determined with a view to ensuring their compliance with those included in the *Jaɓnde* tool. Yet forage nutritional values can vary significantly depending on harvesting stage and storage conditions, leading to potential differences between farms. In addition, live weight, a key metric in determining cows' nutritional requirements, was estimated using a weighing tape, which is not particularly accurate. As a result, cows' requirements may have been underestimated or overestimated.

According to dairy farmers, several constraints may have contributed to some cows failing to meet their milk production targets, such as fodder refusals and poor fodder quality, low water availability, low pasture quality, and long distances travelled. These constraints have been shown by several authors to limit livestock productivity (Diawara et al., 2017; Ouattara et al., 2024; Sib et al., 2017; Sodré et al., 2022). Furthermore, the varying quality of milking practices implemented by herdsmen, combined with aggressive or stressful behaviour displayed by some of the cows during milking, may have influenced the actual production potential of some cows. Similarly, in Benin, Kassa et al. (2016a) and Sissao et al. (2018) found that cow milk production was strongly influenced by milking intensity.

Others methodological limits could be pointed. Although the Dairy Innovation Platform includes many of the main dairy farmers in the Bobo-Dioulasso milkshed area, voluntary participation may have introduced a selection bias. Participating dairy farmers may have been more open to innovation than the broader dairy farming population. Therefore, caution is required when extrapolating the findings to all dairy farmers in Burkina Faso or other sub-Saharan African contexts. The study design did not include a randomised or counterfactual comparison group; consequently, the share of the found gains that stems specifically from the tool, as opposed to concurrent changes in feeding effort or husbandry, remains difficult to isolate. Also, the monitoring window was short, even it generally matches with most of feeding trial design in station (14-day adaptation phase follow by a 7-day data collection period; Somda et al. (2025)). In real condition, outcomes that unfold over a full lactation, such as the evolution of body condition, the persistency of the lactation curve, animal health, or farm-level profitability over time, fall outside the scope of what could be measured here. Finally, because each participating farm contributed more than one cow, the observations are unlikely to be fully independent; this nested structure was not modelled, and the resulting within-farm correlation may have led the precision of the estimates to be somewhat overstated. In addition, the cows included in the trial were selected by the dairy farmers, which may have introduced a selection bias and limited the representativeness of the sampled cows.

Lastly, the results may have been influenced by the trial's environmental variability and dairy farmers' practices.

This study also has several major strengths. It was conducted under real smallholder dairy farming conditions in West Africa, which enhances the relevance and applicability of the findings to smallholder farming systems, as opposed to the controlled conditions of experimental stations.

The study included both zebu and crossbred cows, thereby reflecting the genetic diversity of local dairy production systems. It also combined nutritional, economic, and environmental variables, including feed intake, nutrient supply, ration costs, gross margin, enteric methane emissions, and farmer satisfaction, thus providing a multidimensional assessment of performance. Another strength of the study lies in the adaptation of *Jaɓnde* to local conditions, particularly through the consideration of tropical forage resources, grazing practices, genetic types, climatic constraints, and the management realities faced by farmers.

The participatory process used for ration design and for assessing the *Jaɓnde* tool with farmers helped to ensure that the predicted rations were aligned with local practices and facilitated the acceptability of the tool among users.

Finally, the results provide a framework for the preliminary field validation of a ration formulation tool and constitute a solid basis for future large-scale studies encompassing multiple seasons and different agroecological zones.

## 5 Conclusion

This study provides a preliminary field validation of *Jaɓnde* tool, whose outcomes can be read along three lines.

First, the comparison of cow types confirmed the higher absolute productivity of crossbred cows but a greater relative response in zebu cows: average milk production was 10.6 L/d/cow among crossbred cows, corresponding to an 8% increase, compared with 1.2 L/d/cow for zebu cows, which corresponds to a 33% increase.

Second, dairy farmers implemented actual ration (R3) close to those predicted (R2) with mean differences of −0.10, −0.37, and +0.04 kg GM/d/cow for quality fodder, coarse fodder, and feed concentrates; demonstrating good implementation fidelity. The tool showed encouraging though still preliminary predictive performance with limited systematic bias: MBE ≤ −0.15 kg GM/d/cow for ingested rations; MBE= −0.27 L/d/cow for milk production, and −99.98 F CFA/d/cow for gross profit margin. *Jaɓnde* appears to be less effective in crossbred cows in terms of milk production.

Third, satisfaction was high, with >90% of participating dairy farmers reporting perceived gains in milk production and expressing satisfaction with the tool. *Jaɓnde* least-cost formulation logic is nonetheless directly relevant where feed prices are volatile, and its integration of ambient temperature makes it pertinent under increasing climate variability.

Before recommending the tool for wider use by livestock advisors, its robustness should be confirmed through external validation in independent dairy farming populations across a wider range of farms, seasons, feed-supply situations, agroecological zones, and management systems, thereby establishing its scalability beyond the present study area.

## Consent to participation

Informed consent was obtained from dairy farmers before the experimentation and interview. They consented to the submission of data for publication.

## Ethics approval

The research focuses exclusively on the observation of animal feeding practices on farms, as part of field studies. No animal handling, biological sampling (blood, tissue, etc.), or restrictive measures (caging, isolation, etc.) are involved. This work is fully respectful of animal welfare, without inducing stress, suffering, or deleterious effects on the animals concerned.

## Generative AI statement

The author(s) declare that no generative AI was used in the creation of this manuscript.

## Funding

The author(s) declare that financial support was received for the research of this article. This research was funded by the CGIAR Initiative on Agroecology (INIT-31; Biodiversity-A1562 & CIAT-G193) under the CGIAR Research Portfolio supporting the 2030 Sustainable Development Goals.

## CRediT authorship contribution statement

**Songdah Désiré Ouattara:** Writing – review & editing, Writing – original draft, Visualization, Methodology, Investigation, Formal analysis, Data curation, Conceptualization. **Ollo Sib:** Writing – review & editing, Validation, Supervision, Resources, Project administration, Methodology, Funding acquisition, Conceptualization. **Mohamed Habibou Assouma:** Writing – review & editing, Resources, Methodology. **Souleymane Sanogo:** Writing – review & editing, Validation, Methodology, Investigation. **Beh Traoré:** Writing – review & editing. **Vinsoun Millogo:** Writing – review & editing. **Valérie Marie Christiane Bougouma-Yaméogo:** Writing – review & editing, Validation, Supervision, Conceptualization. **Eric Vall:** Writing – review & editing, Validation, Supervision, Resources, Project administration, Methodology, Funding acquisition, Conceptualization.

## Declaration of competing interest

The authors declare that the research was conducted in the absence of any commercial or financial relationships that could be construed as a potential conflict of interest.
